# The innate memory response of macrophages to *Mycobacterium tuberculosis* is shaped by the nature of the antigenic stimuli

**DOI:** 10.1128/spectrum.00473-24

**Published:** 2024-07-09

**Authors:** Ranjeet Kumar, Afsal Kolloli, Pooja Singh, Lanbo Shi, Andreas Kupz, Selvakumar Subbian

**Affiliations:** 1 Public Health Research Institute, Rutgers-New Jersey Medical School, Newark, New Jersey, USA; 2 Centre for Molecular Therapeutics, Australian Institute of Tropical Health and Medicine (AITHM), James Cook University, Cairns & Townsville, Queensland, Australia; National Center for Biological Sciences, Bangalore, Karnataka, India

**Keywords:** trained immunity, tuberculosis, autophagy, immunometabolism, inflammation

## Abstract

Innate immune cells, such as macrophages, mount an immune response upon exposure to antigens and pathogens. Emerging evidence shows that macrophages exposed to an antigen can generate a “memory-like” response (a.k.a. trained immunity), which confers a non-specific and enhanced response upon subsequent stimulation with a second antigen/microbe. This trained immunity has been implicated in the enhanced response of macrophages against several invading pathogens. However, the association between the nature of the antigen and the corresponding immune correlate of elicited trained immunity is not fully understood. Similarly, the response of macrophages trained and restimulated with homologous stimulants to subsequent infection by pathogenic *Mycobacterium tuberculosis* (Mtb) remains unexplored. Here, we report the immune and metabolic profiles of trained immunity in human THP-1-derived macrophages after homologous training and restimulation with BCG, LPS, purified protein Derivative (PPD), heat-killed Mtb strains HN878 (hk-HN), and CDC1551 (hk-CDC). Furthermore, the impact of training on the autophagic and antimicrobial responses of macrophages with or without subsequent infection by clinical Mtb isolates HN878 and CDC1551 was evaluated. Results show that repeated stimulation of macrophages with different antigens displays distinct pro-inflammatory, metabolic, antimicrobial, and autophagy induction profiles. These macrophages also induce a differential antimicrobial response upon infection with clinical Mtb HN878 and CDC1551 isolates. A significantly reduced intracellular bacterial load was noted in the stimulated macrophages, which was augmented by the addition of rapamycin, an autophagy inducer. These observations suggest that the nature of the antigen and the mode of stimulation shape the magnitude and breadth of macrophage innate memory response, which impacts subsequent response to Mtb infection.

**IMPORTANCE:**

Trained immunity (a.k.a. innate memory response) is a novel concept that has been rapidly emerging as a mechanism underpinning the non-specific immunity of innate immune cells, such as macrophages. However, the association between the nature of the stimuli and the corresponding immune correlate of trained immunity is not fully understood. Similarly, the kinetics of immunological and metabolic characteristics of macrophages upon “training” by the same antigen as primary and secondary stimuli (homologous stimulation) are not fully characterized. Furthermore, the ability of antigens such as purified protein derivative (PPD) and heat-killed-Mtb to induce trained immunity remains unknown. Similarly, the response of macrophages primed and trained by homologous stimulants to subsequent infection by pathogenic Mtb is yet to be reported. In this study, we evaluated the hypothesis that the nature of the stimuli impacts the depth and breadth of trained immunity in macrophages, which differentially affects their response to Mtb infection.

## INTRODUCTION

The primary host defense against bacterial infections is driven by innate immune cells such as macrophages and neutrophils. Though innate immune cell responses are rapid, they are non-specific in their effector functions following infection (1, 2). Innate immune receptors, namely the pattern recognition receptors (PRRs) present on the host cell surface recognize a multitude of evolutionarily conserved molecules shared by a plethora of infectious agents (3, 4). Engagement of different types of PRRs, such as Toll-like receptors (TLRs), with various microbial components, including lipopolysaccharides (LPS), β-glucan, non-methylated CpG, and double-stranded RNA can mount remarkable changes in the immunometabolic profiles of phagocytes and their antimicrobial responses upon subsequent bacterial and viral infections (5–7).

Immunological memory has traditionally been considered an attribute of the host adaptive immune system, constituted mainly by T and B cells. However, recent studies have reported that innate immune cells, such as macrophages can generate a “memory-like” response upon antigen stimulation and/or exposure to microbes (8, 9). These “trained” cells respond rapidly and robustly to subsequent stimulation with diverse antigens or pathogens (8, 9). This de facto innate immune memory response has been termed “trained immunity” (10). Notably, trained immunity developed in innate immune cells is a special feature because of cell activation by a stimulant. The cellular behavior toward various stimulations and/or infections is exceptionally diverse, sharing some similarities and differences between trained immunity and other immune processes. Depending on the stimulation niche, the innate immune cells demonstrate substantial plasticity and adaption. It is important to note that the magnitude (low versus high dose) and duration (short versus long) of stimulation induce specific adaptations in innate immune cells that reflect their requirement to either enhance immune responses or prevent immunity and excessive immunopathology. Several such adaptive programs, including cell differentiation, priming, tolerance, and trained immunity, have been described in innate cells (5–10). Vaccination of infants with BCG has been cited as a prototypic example of this concept, wherein innate cells exposed to BCG were “trained” and helped in the protection against not only tuberculosis (TB), the intended target of BCG vaccination but also against other unrelated infectious diseases, including yellow fever and malaria (11–17). Epidemiological data suggest that live vaccines such as the measles, smallpox, and oral polio vaccines can also confer host-protective effects against non-targeted pathogens through trained immunity (18). *In vitro*, macrophages, neutrophils, and natural killer (NK) cells as well as non-immune cells, such as fibroblasts, have been shown to exert trained immunity phenotype upon stimulation with BCG, LPS, or β-glucan; in general, the training involves a primary stimulation with the antigen for 24 h followed by a resting period of about 3–6 days and a second stimulation with a heterologous antigen (19–24). In human primary monocytes primed with β-glucan, BCG, and oxLDL (oxidized low-density lipoprotein), elevated cytokine production was noted after 3-to-6 days post-primary stimulation (25). However, studies conducted in mice and humans suggest that the effects of trained immunity were retained for 3 months to 5 years post-antigen stimulation and/or vaccination (26).

In various innate cells, stimulation with BCG or viral or fungal cell wall components elicits a trained immunity phenotype, marked with elevated expression of TNF-α and IL-6 (27). While trained PMNs induce IL-8 and reactive oxygen species (ROS), mTOR, and lactate production, ILCs induce IL-5 and IL-13, and NK cells express higher levels of IL-1β and IFN-γ, compared to non-trained cells (27). Importantly, epigenetic modifications involving H3K4 monomethylation (H3K4me), trimethylation (H3K4me3), and H3K27ac are the underlying regulatory mechanism of trained immunity in these cells, which manifests in enhanced proinflammatory response, antigen processing, and presentation as well as elevated antimicrobial responses (27). Of note, training of monocytes/macrophages differs from priming/activation in that the primed cells might be re-stimulated before the gene transcription induced by the initial stimulus subsides, which will enhance their inflammatory response; while trained monocytes/macrophages maintain long-term epigenetic changes at specific gene promoters after the immune activation status returns to baseline as demonstrated in previous studies (5–10). Cumulatively, the onset of trained immunity, such as during BCG vaccination, not only exerts a robust innate response but also confers non-specific cross-protection against several pathogens (28).

Induction of proinflammatory cytokine/chemokine production such as TNF-α, IL-6 and IL-1β, elevated glycolysis and autophagy, and increased antimicrobial responses are among the top biomarkers of trained immune response (19, 23, 29). In a recent study by Zhang et al., the development of heterogeneous trained immunity in human monocytes upon training with different inducers was studied (30, 31). However, the kinetics of immunological and metabolic characteristics of macrophages upon “training” and restimulation (boosting) by the same antigen are not fully explored. Similarly, most of the trained immunity studies in macrophages were conducted using BCG, LPS, and β-glucan (19–24, 29). However, in the context of TB, the ability of other antigens such as PPD (a.k.a. tuberculin) and hk-Mtb to induce trained immunity in macrophages gains importance (32). However, the nature of the immune response and subsequent effector functions elicited by repeated stimulation with these antigen cocktails in macrophages remains unexplored.

Pathogenic Mtb strains show diverse genetic components and induce heterogeneous cellular and host responses in infected humans (33–36). For example, Mtb lineage 2/W-Beijing strains are associated with an increased number of active and drug-resistant TB cases (37, 38). These strains trigger a strong inflammatory response upon macrophage phagocytosis, due in part to the variation in their cell surface molecules, such as ManLAM and phenolic glycolipids (PGL) (39, 40). Epidemiological and pre-clinical studies have shown a minimal protective effect of BCG vaccination against infections caused by the lineage 2 Mtb strains (39, 41–43). By contrast, Mtb strains belonging to the Euro-American Lineage, lineage 4, that lack PGL are susceptible to engulfment by phagocytes and are controlled better by the host immunity elicited by BCG vaccination (44–46). Studies with *in vitro* and *in vivo* models of TB have also suggested differential outcomes of infection based on the lineage of Mtb strains (47–53). Importantly, the immune responses of macrophages trained with different stimuli and their impact on the outcome of subsequent infection with different clinical Mtb isolates remain unknown.

In this study, we evaluated the hypothesis that the nature of the antigen and the mode of stimulation (training and restimulation) impact the depth and breadth of trained immunity in macrophages, which differentially affects their response to Mtb infection. To test this hypothesis, we performed a comprehensive analysis of the immunological, metabolic, and anti-microbial responses of human macrophages after training, followed by restimulation/boosting with BCG, LPS, PPD, hk-HN, or hk-CDC, and with or without subsequent infection with clinical Mtb isolates HN878 or CDC1551. Our findings indicate that the nature of the antigen used for stimulation significantly and differentially affects the immune profile of trained/restimulated macrophages and their subsequent response to Mtb infection.

## MATERIALS AND METHODS

### Chemicals and reagents

Unless specified otherwise, all chemicals and reagents used in this study were purchased from MilliporeSigma (Sigma-Aldrich, Inc., St. Louis, MO, USA). Gene-specific primers were synthesized at Integrated DNA Technologies (IDT, Coralville, IA, USA). LPS from *E. coli* O111:B4 was purchased from InvivoGen (San Diego, CA, USA) and used as we reported previously (54). BCG-Tice strain (BCG) was from Organon Teknika Corporation LLC (Durham, NC, USA), and PPD was obtained from the Staten Serum Institut, Denmark, and used as described previously (55). To generate heat-killed HN878 (hk-HN) and CDC1551 (hk-CDC) strains, mid-log phase cultures were pelleted at 5,000 rpm for 10 minutes at 4°C, and the pellets were washed with sterile 1× PBS and resuspended thoroughly in the same buffer. The bacterial cultures were boiled for 45 minutes, cooled and the total protein content was measured using the BCA Protein Assay Kit (Thermo Fisher Scientific, Waltham, MA, USA).

### Human macrophage cell line

The human monocytic cells (THP-1) were obtained from ATCC (American Type Culture Collection, Manassas, VA, USA) and grown in RPMI media supplemented with 10% FBS and L-glutamine (complete RPMI; Sigma, St, Louis, MO, USA). The cells were grown in incubators set at 37°C with 5% CO_2_. Once in 2–3 days or when the cells reached about 75% confluency, they were either passaged or used for experiments. To maintain consistency between experiments, cells were seeded at about 80% confluency for each experiment. Cells were checked for contamination by microscopic examination. The viability of cells was determined by microscopic analysis of cell suspension stained with 4% trypan blue solution on a hemocytometer slide. All assays were performed with cells having >90% viability without any contamination. The cells were treated with 40 nM phorbol 12-myristate 13-acetate (PMA) (Sigma, St, Louis, MO, USA) for 24 h in a complete RPMI medium, to differentiate them into macrophages, washed and incubated further in fresh media for 24 h before conducting the experiments.

### *Mycobacterium tuberculosis* culture conditions

Low-passaged laboratory stocks of clinical Mtb isolates HN878 and CDC1551 and strain H37Rv were cultured in Middlebrook 7H9 liquid media supplemented with 10% ADC (Difco BD, Franklin lakes, NJ), 5% glucose, and 0.05% tween 80 under continuous shaking at 37°C as reported previously (56). Mid-log-phase cultures with OD_600_ of 0.4 to 0.6 were harvested and 1 mL aliquot was stored at –80°C until further use. These stocks were evaluated for the number of bacterial colony-forming units (CFU) by plating serial dilutions of multiple stock vials on Middlebrook 7H10 agar plates (Difco BD, Franklin lakes, NJ), supplemented with 5% glucose, 10% oleic acid, albumin, dextrose, and catalase, and 0.05% tween 80 and incubating for 4–6 weeks at 37°C before enumerating colonies. The bacterial load was expressed as CFU/mL. All experiments involving live Mtb cultures were performed in biosafety level-3 (BSL3) facilities as per the protocols approved by the Rutgers University Institutional Biosafety Committee (IBC).

### *In vitro* training and restimulation of macrophages

THP-1-derived macrophages were trained with different antigenic stimuli as previously described and shown in Fig. S1A (15, 57). Briefly, cells were stimulated for 24 h (first stimulation) with BCG-Tice strain at a multiplicity of infection (MOI) of 10, LPS (100 ng/mL/well), PPD (100 ng/mL/well), and hk-CDC or hk-HN (5 µg/mL/well). Macrophages without any stimulation were used as a negative control. After 24 h of the first stimulation, cell-free supernatants and cell pellets were collected separately to determine the primary response (i.e., after the first stimulation). In an identical setting, macrophages were washed after the first stimulation and maintained/rested in complete RPMI media for 4 days for training. On day 5, macrophages were restimulated (second stimulation) for 24 h with the same antigens as the primary stimulation (see above). After the first and second stimulation, cell-free supernatants of stimulated and non-stimulated macrophages were collected, filtered through a 0.45-µm filter (VWR International, Radnor, PA, USA), and used for cytokine estimation. The cell pellets were lysed with TRI reagent as per the manufacturer’s instructions (MRC, Cincinnati, OH, USA) for RNA isolation (see below).

### Mtb infection studies

THP-1-derived macrophages were trained/restimulated with various stimuli as above, followed by infection with Mtb HN878 or CDC1551, as shown in Fig. S1B, at an MOI of 1 for 24 h at 37°C with 5% CO_2_ supply as described previously (58). Cell-free supernatant was collected, filtered as above, and used for cytokine analysis. Cell pellets were washed once with 1× PBS and lysed either with TRI reagent (for macrophage RNA isolation) or 1/10 vol of 0.5% Triton X-100 solution to determine intracellular Mtb load. For Mtb CFU analysis, infected macrophages in Triton X-100 were probe-sonicated as 10 pulses of 10 seconds each with intermittent ice incubations for 30 seconds. Lysed cells were serially diluted in 1× PBS and plated on Middlebrook 7H10 agar plates to determine the number of bacterial CFU as mentioned above.

### Visualization of Mtb in infected macrophages

The auramine-Rhodamine staining kit (Hardy Diagnostics, Santa Maria, CA, USA) was used according to the manufacturer’s instructions to visualize Mtb in infected macrophages. Briefly, macrophages were seeded on glass coverslips inside a 24-well plate and infected with Mtb at MOI of 1 for 3 hours. Uninfected macrophages were used as controls. The Mtb-infected and uninfected macrophages were fixed overnight with 10% formalin solution, followed by washing five times with sterile 1× PBS and incubation in 70% ethanol overnight. The macrophages were stained with Auramine-Rhodamine solution for 15 minutes, followed by rinsing with water, and 0.5% acid-alcohol for 3 minutes each. The cells were rinsed with water and counter-stained with 1% KMnO_4_ solution for 2 minutes. The coverslips with stained cells were gently removed, air-dried, mounted on glass slides, and examined under a fluorescence microscope using a 63× objective.

### Macrophage RNA extraction and quantitative PCR assay

Total RNA from THP-1-derived macrophages was isolated from stimulated and non-stimulated cells with or without Mtb infection, using the TRI reagent method, as per the manufacturer’s instructions (Molecular Research Center Inc, Cincinnati, OH, USA). The RNA was purified using the Qiagen RNeasy kit as per the manufacturer’s instructions (Qiagen Inc, USA), and quantified in a NanoDrop instrument (ThermoFisher Scientific, Waltham, MA, USA). For cDNA synthesis, 1 µg of RNA was reverse transcribed using a Superscript III kit as per the manufacturer’s protocol (Thermo Fisher Scientific, Waltham, MA, USA). Real-time qPCR was performed using PowerTrack SYBR Green Master Mix (ThermoFisher Scientific, Waltham, MA, USA). Primers for target host genes were designed using the Primer3 software (NIH, Bethesda, USA), and β-actin (*ACTNB*) gene transcript levels were used to normalize the expression of target gene transcripts in each test sample. Relative fold change in gene expression was calculated by the 2^−ΔΔCt^ method as reported previously (34). The DNA sequence of forward and reverse primers of target genes used in qPCR analysis is shown in Table S1.

### Multiplex cytokine assay

Cytokines in the culture supernatants were analyzed using the Human Cytokine Magnetic 10-Plex Panel (ThermoFisher Scientific, Waltham, MA, USA) following the manufacturer’s instructions. Briefly, antibody beads from the kit were diluted to 1×, and 25 µL was added in each well of a 96-well plate, followed by the addition of culture supernatants or standard. Then the plate was incubated at 4°C overnight. The wells were washed twice and 100 µL of Biotinylated Detector Antibody was added and the plate was incubated at room temperature for an hour. After washing twice, 100 µL of 1× Streptavidin-RPE was added, and the plate was incubated for 30 minutes. Finally, the plate was washed twice and read in the Luminex System. The data were analyzed using ProcarataPlex Analysis software (ThermoFisher Scientific, Waltham, MA, USA). The data were collected from two independent experiments.

### Nitric oxide estimation

The macrophage nitric oxide (NO) production was estimated by measuring nitrite levels using a Nitric Oxide Colorimetric Assay Kit following the manufacturer’s instructions (Enzo Life Sciences, Farmingdale, NY, USA) as described previously (59). Briefly, culture supernatants from trained and restimulated, and untrained macrophages with or without subsequent Mtb infection were filtered through 0.45 µm filters and used for NO estimation. Filtered culture supernatant (25 µL) and serial dilutions of sodium nitrite standard and control (complete RPMI) were added to a 96-well plate along with the kit reagents. Samples were mixed gently, and the plate was incubated for 30 minutes at 37°C. Post-incubation, 50 µL of Griess reagent was added to each well and further incubated at room temperature for 10 minutes in the dark. The plate was read at OD_540-570_ nm using a GloMax spectrophotometer (Promega, Madison, WI, USA). Optical density readings were plotted on a standard curve graph prepared using dilutions of known concentrations of sodium nitrite. NO concentrations of unknown test samples were calculated using the extrapolation of the standard curve.

### ATP and lactate production assay

To assess the metabolic milieu of trained restimulated macrophages, particularly on glycolysis or oxidative phosphorylation after Mtb infection, the production of ATP and lactate was measured using Glycolysis/OXPHOS Assay Kit as per the manufacturer’s instructions (Dojindo Molecular Technologies, Rockville, MD, USA). In this assay, oligomycin and 2-DG inhibit ATP synthesis by oxidative phosphorylation and glycolysis, respectively. Briefly, after the second stimulation/boosting, macrophages were seeded at a density of 1 × 10^4^ cells in each well of a 96-well black plate and incubated overnight at 37°C maintaining 5% CO_2_. The following day, cells were infected with Mtb CDC1551 or HN878 at an MOI of 1. After 24 h, infected and non-infected (controls) macrophages were incubated in a medium containing 1.25 µM oligomycin for 5 h to inhibit mitochondrial oxidative ATP production or 22.5 mM 2-deoxy-D-glucose (2-DG) to inhibit glycolytic ATP production. Total cellular ATP was measured without any drug treatment. The assay plates were incubated at 25°C for 10 minutes in a microplate reader and luminescence was measured as a surrogate for ATP levels. In a similar experiment, total glycolytic and oxphos lactate production was measured using the cell supernatant, which was incubated with the reaction mixture (supplied with the Glycolysis/OXPHOS Assay Kit) at 37°C for 30 minutes, followed by measuring absorbance at 450 nm using the GloMax spectrophotometer (Promega).

### Lactate dehydrogenase activity assay

Lactate dehydrogenase (LDH) release from cells into culture supernatants was detected using the LDH-Glo Cytotoxicity assay kit following the manufacturer’s instructions (Promega). Briefly, the culture supernatants were diluted in LDH storage buffer and 50 µL was added into each well of a 96-well plate. LDH detection reagent was prepared and 50 µL was added to each well. Then the plate was incubated for 60 minutes at room temperature and luminescence was recorded using a GloMax spectrophotometer (Promega). The luminescence data were plotted against a standard curve generated with the known concentration of LDH, and the final values were represented as mUnits/mL of the supernatant (Promega).

### Immunostaining analysis

For immunostaining, macrophages were seeded at a density of 1 × 10^5^ cells in each well of a 24-well plate containing a coverslip within the well. The cells were trained and restimulated with different stimulants and infected with Mtb strains as described above. After infection, the cells were fixed for 20 min with 4% paraformaldehyde in cold PBS, washed three times with 2% BSA (bovine serum albumin) in 1× PBS, treated with 0.05% Triton X-100 in PBS, and incubated overnight at 4°C with the following anti-human primary antibodies at 1:250 dilution in 2% BSA: (i) anti-p62 (H00008878-MO1, ThermoFisher Scientific), (ii) anti-LC3 (NB100-2220AF647, Novus Biologicals, Centennial, CO, USA), (iii) anti-TNFα (NBP1-9532AF532, Novus Biologicals), and (iv) anti-IL-1β (NBP1-19775AF594, Novus Biologicals). Cells were washed three times with 2% BSA and incubated for 1 h with Alexa-Fluor-488-conjugated goat anti-mouse IgG (H&L) secondary antibody (ab150113, Abcam, Waltham, MA, USA) at 1/2,000 dilution. Cells were washed three times with sterile 1× PBS, and the coverslips were mounted on a glass slide using a mounting medium (ab104139, Abcam, Waltham, MA, USA). Images were acquired using an Axiovert 200M inverted fluorescence microscope (Zeiss, Oberkochen, Germany) using 20× or 63× (oil immersion) objective and a Prime sCMOS camera (Photometrics, Tucson, A2) controlled by Metamorph image acquisition software (Molecular Devices, San Jose, CA).

### Statistical analysis

All experiments were conducted in a minimum of three biological replicates, repeated at least twice, and the average value of two technical replicates was used for graphical presentations as the mean ± standard error values. Comparisons between two experimental conditions were analyzed by unpaired *t*-test with Welsh correction, and for multiple group comparison, one-way ANOVA with Tukey’s correction or two-way ANOVA was used. Statistical analysis was performed using GraphPad Prism 9.3 version (GraphPad Software, La Jolla, CA). For all the experimental data comparisons between groups, differences were considered statistically significant when *P* ≤ 0.05.

## RESULTS

### Immune response of THP-1-derived macrophages trained and restimulated with homogeneous antigens

**Fig 1:**
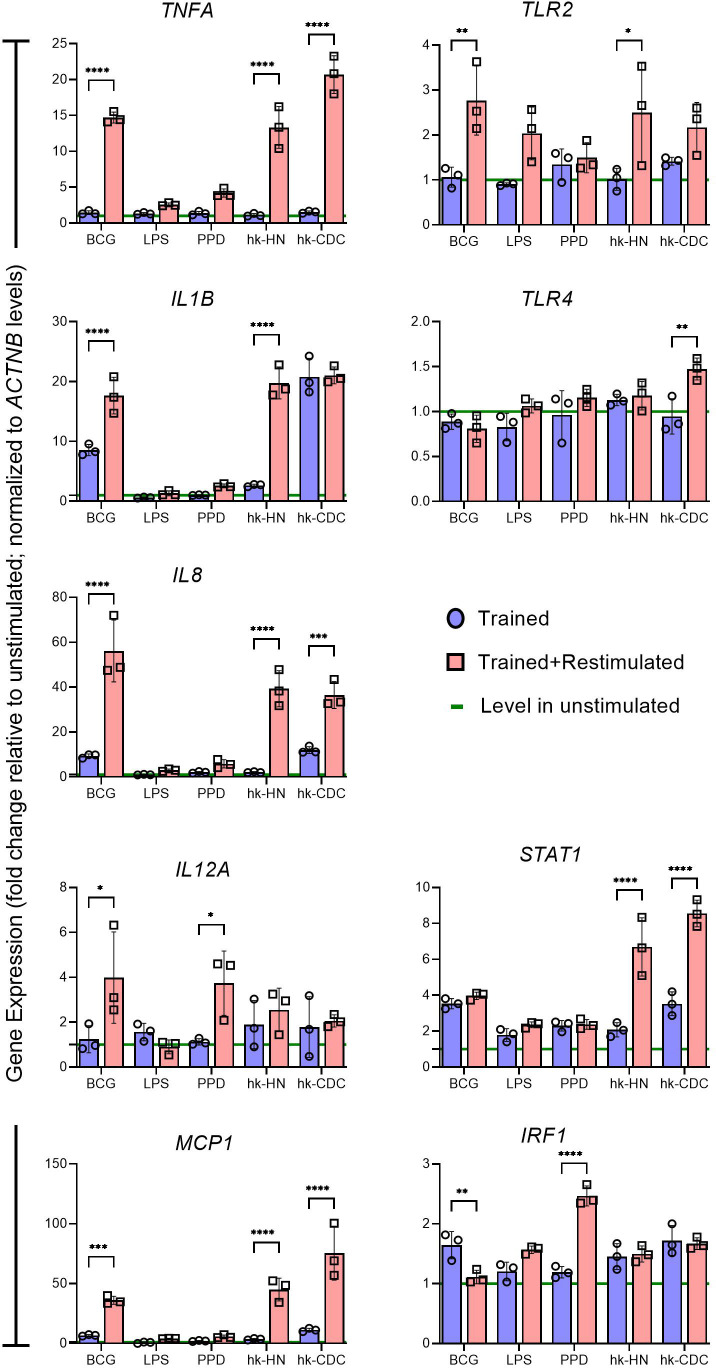
Immune activation marker gene expression profile of uninfected human macrophages trained and restimulated with various antigens. THP-1-derived macrophages were stimulated with BCG, LPS, PPD, hk-HN878, or hk-CDC1551 on day 1 (trained cells) and day 6 (trained and restimulated cells) as described in the methods section. At both time points, total RNA was isolated, and real-time qPCR analysis was performed to quantitate the expression of genes encoding proinflammatory cytokines/chemokines (TNF-α, IL-1β, IL-8, IFN-γ, IL-12A, and MCP-1), cell surface/nuclear receptors (TLR2 and TLR4), and transcriptional regulators (STAT1 and IRF1). RPMI refers to cell-free supernatant of trained macrophages without restimulation (control). Target gene expression was normalized to the *ACTNB* expression levels in corresponding samples. The expression level of each test gene in the trained/restimulated samples was calibrated to the control (RPMI), and the values are shown as relative gene expression. The data shown are the average of three independent experiments performed twice, and the average of three experiments was used for plotting the graph. Statistical analyses were performed using one-way ANOVA with Tukey’s multiple-group comparison. *P < 0.05; **P < 0.01; ***P < 0.005; ****P < 0.001.

**Fig 2:**
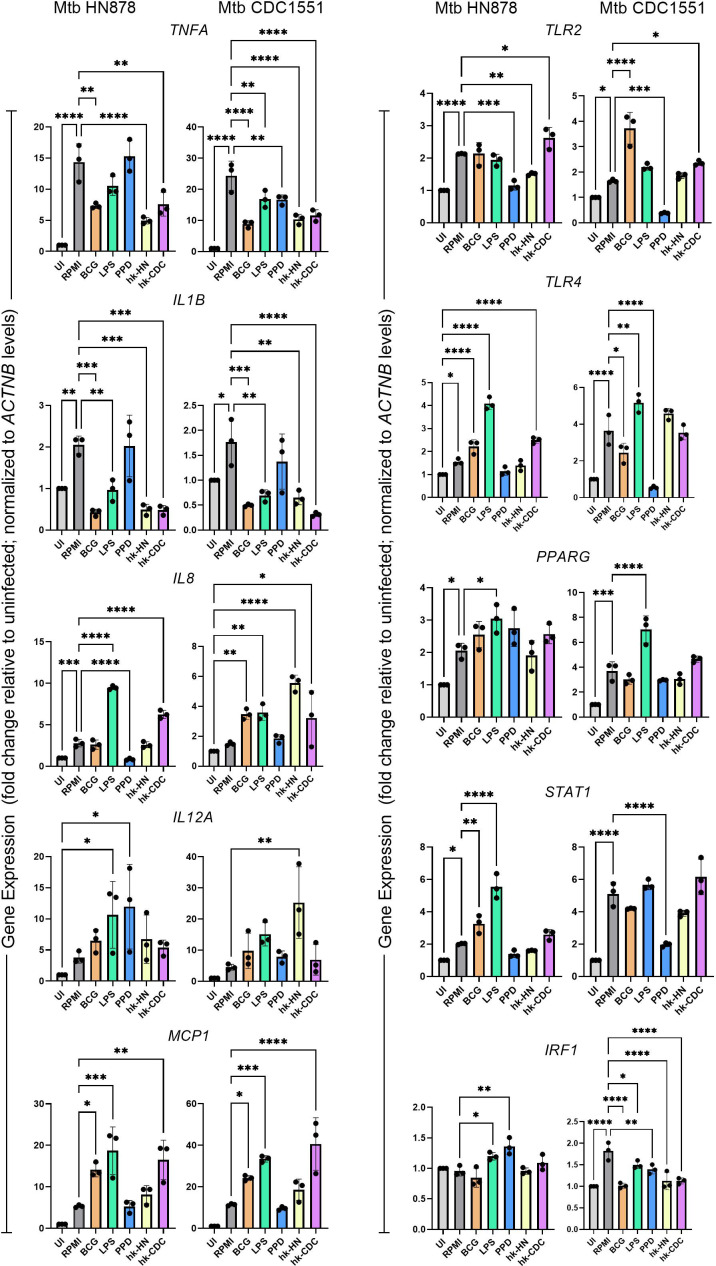
Expression profile of immune activation markers in trained and restimulated macrophages after infection with clinical Mtb isolates. THP-1-derived macrophages were trained and restimulated with BCG, LPS, PPD, hk-HN878, or hk-CDC1551, and infected with clinical Mtb isolates, HN878 or CDC1551 for 24 h. Total RNA was isolated and used for qPCR analysis of genes encoding cytokines/chemokines (TNF-α, IL-1β, IL-8, IFN-γ, IL-12, and MCP-1), cell surface/nuclear receptors (TLR2, TLR4, and PPAR-γ) and transcriptional regulators (STAT1 and IRF1). RPMI refers to cell-free supernatant of trained macrophages without restimulation (control). Target gene expression was normalized to the *ACTNB* expression levels in corresponding samples. The expression level of each test gene in the trained/restimulated samples was calibrated to the control (RPMI), and the values are shown as relative to uninfected samples. The data shown are the average of three independent experiments performed twice, and the average of three experiments was used for plotting the graph. Statistical analyses were performed using one-way ANOVA with Tukey’s multiple-group comparison. *P < 0.05; **P < 0.01; ***P < 0.005; ****P < 0.001.

PMA-differentiated THP-1 cells have been extensively used as a surrogate to study human macrophage functions, including immunological and metabolic signaling analysis upon antigen stimulation (60–62). The immune response of macrophages after the induction of trained immunity, by exposure to different/divergent antigens (e.g., first exposure with BCG and second exposure with LPS) has been reported in several studies (63–66). However, trained immunity developed in macrophages after restimulation to the same (homogeneous) antigen has not been fully evaluated. Here, we used BCG, LPS, and other cocktail antigens relevant to TB, including PPD, hk-HN, and hk-CDC as both primary and secondary stimulants and determined the expression of immune activation marker genes by qPCR (Fig. 1 and 2). We did not find any difference in cell viability between unstimulated and stimulated and/or restimulated macrophages with or without Mtb infection (until 24 h post-infection). First, to determine the immune response of trained macrophages to various test antigens, the expression of proinflammatory markers (*TNFA*, *IL1B*, *IL8*, *IL12A,* and *MCP1*), cell surface receptors (*TLR2* and *TLR4*) and transcriptional regulators (*STAT1* and *IRF1*) was measured after 24 h of first stimulation (Fig. 2). Among these genes, expression of *TNFA*, *IL1B*, *IL8*, *IL12A*, *STAT1,* and *IRF1* was significantly upregulated in BCG and hk-CDC-trained macrophages, compared to unstimulated cells. Expression of *IRF1* was significantly upregulated in hk-HN-stimulated cells, while significant upregulation of *STAT1* expression was noted in cells exposed to all stimulants, except for LPS, which showed an upregulation that was not significantly different from unstimulated cells. Thus, a differential immune profile, impacting cytokine, chemokine, and surface receptor expression, was noted in THP-1 macrophages after 24 h of stimulation with various antigens.

Next, we determined the immune activation status of these trained macrophages to restimulation by measuring the expression pattern and directionality of selected genes as above (Fig. S1A). The level of induction of several of the tested genes was higher in the trained cells after restimulation with the same antigens, compared to no restimulation (Fig. 1). Notably, restimulation with BCG significantly upregulated the expression of all tested genes except for *TLR4* and *STAT1*, while the expression of *TNFA*, *IL8, MCP1,* and *STAT1* was significantly upregulated upon restimulation with hk-HN or hk-CDC, while restimulation with BCG downregulated IRF1 expression. In addition, the expression of *IL12A* and *IRF1* was significantly upregulated in PPD-stimulated trained macrophages. Expression of *TLR2* and *TLR4* was upregulated by hk-HN and hk-CDC-treated trained cells, respectively (Fig. 1). We observed a higher expression of IL-1β in hk-HN trained and restimulated macrophages compared to trained cells, while training and restimulation with hk-CDC in macrophages resulted in similar expression levels of IL-1β. One possible reason we envisage specifically regarding IL-1β expression among trained and trained + restimulated cells for hk-CDC-treated cells is that CDC1551 is known to induce hyperimmunogenicity as reported in those previously published studies. So even the first stimulation rendered cells to express a higher level of IL-1β expression. However, additional studies are needed to confirm this notion.

We validated the gene expression profile of trained and restimulated macrophages by measuring the protein levels of respective immune activation markers in the culture supernatant of macrophages by ELISA (Fig. S2). Consistent with the qPCR results, the protein levels of immune activation markers, TNF-α, IL-1β, IL-6, and GM-CSF, as well as immune modulators IL-4 and IL-10 were upregulated to various extents in the macrophages trained and restimulated with different stimulants, compared to only first stimulation. Furthermore, the levels of IFN-γ, IL-2, and IL-5 were not impacted by the restimulation of trained macrophages (Fig. S2). Together, these observations strongly suggest that upon restimulation with homogeneous antigen, the trained macrophages are endowed with a unique, antigen-dependent immune activation profile, which was more robust than the primary stimulation with the same antigens.

### Response of trained and restimulated THP-1 macrophages to Mtb infection

Since the restimulation of trained macrophages with various antigens elicited a stronger stimulant-dependent immune activation profile, we hypothesized that these restimulated macrophages would respond differentially than the trained cells to subsequent Mtb infection. We used clinical Mtb isolates, HN878 and CDC1551 to infect trained macrophages with or without restimulation with BCG, LPS, PPD, hk-HN, or hk-CDC and measured the expression of marker genes of trained immunity by qPCR and compared to the levels in uninfected macrophages (Fig. S1B). The clinical isolate Mtb HN878 belongs to a “hyper-virulent” W-Beijing lineage strain, while Mtb CDC1551 was deemed a “hyper immunogenic” strain in preclinical studies (34, 55, 67–74). Furthermore, these two Mtb strains displayed differential pathogenic potential *in vitro* and animal models (67–74). We used an Mtb MOI of 1 to infect the macrophages to avoid necrosis of macrophages upon infection with hyper-virulent clinical Mtb strain, HN878. At this MOI, we could easily detect Mtb-infected macrophages using auramine-rhodamine staining (Fig. S3). We did not evaluate the differential phagocytosis of these two Mtb strains by the macrophages. As shown in Fig. 2, the overall directionality of expression of several tested host immune genes was comparable between Mtb HN878 and Mtb CDC1551-infected trained/restimulated macrophages, although there were differences noted in the expression level of individual genes, depending on the nature of antigen (Fig. 2). Although trained and restimulated macrophages had elevated immune activation markers production (Fig. 1), Mtb infection of these cells did not further induce the expression of *TNFA*, *IL1B*, *IL8*, *TLR2,* and *IRF1* upon restimulation with most of the stimulants. However, differences in the expression pattern of *IL12A*, *MCP1*, *TLR4*, *PPARG,* and *STAT1* were noted between trained and restimulated macrophages infected with Mtb HN878 and CDC1551. While Mtb CDC1551 infection significantly upregulated the expression of *IL8*, expression of *TLR4, PPARG,* and *STAT1* was significantly upregulated in Mtb HN878-infected trained and restimulated macrophages, compared to the cells without restimulation. While *TNFA*, *IL1B,* and *TLR2* expression was significantly dampened upon both Mtb HN878 and Mtb CDC1551 infection, expression of *MCP1* was significantly upregulated by these infections in macrophages trained and restimulated with BCG, LPS, or hk-CDC. Significant downregulation of *IRF1* was noted in Mtb CDC1551-infected trained/restimulated macrophages, compared to the cells without restimulation (Fig. 2).

To assess the significance of restimulation, we used trained macrophages without restimulation for Mtb infection and measured the expression of proinflammatory cytokines (*TNFA*, *IL-1B*, and *IL8*), chemokine (*MCP-1*) and transcription factor (*STAT1*) in trained and infected macrophages (Fig. S4). For this experiment, THP-1-derived macrophages were treated with stimulants for 24 h followed by resting for 4 days. On day 5, the trained macrophages were infected either with Mtb HN878 or CDC1551, and the total host RNA was used for qPCR analysis. We observed significantly increased expression of TNF-α, IL8, MCP, and STAT1 genes in trained macrophages upon infection by both the Mtb strains, similar to those observed in trained and restimulated macrophages (Fig. S4). However, expression of *IL1B* was significantly reduced in trained macrophages upon mycobacterial infection (Fig. S4). This result suggests that restimulation of trained cells differentially affects the expression of immune activation markers upon subsequent Mtb infection.

Consistent with the qPCR data, the protein measurement of TNF-α and IL-4 by ELISA also strongly suggests the dampening of immune activation in trained and restimulated macrophages upon Mtb infection (Fig. S2). Together, these findings indicate that the diversity in the immune response of trained and restimulated macrophages elicited by various stimulants differentially affects the infection with Mtb HN878 or CDC1551 strains, which differ in their virulence. Thus, both the nature of the antigen and of the infecting Mtb bacteria determine the outcome of infection of trained macrophages.

### Spatial expression of immune activation markers in trained and restimulated macrophages

**Fig 3:**
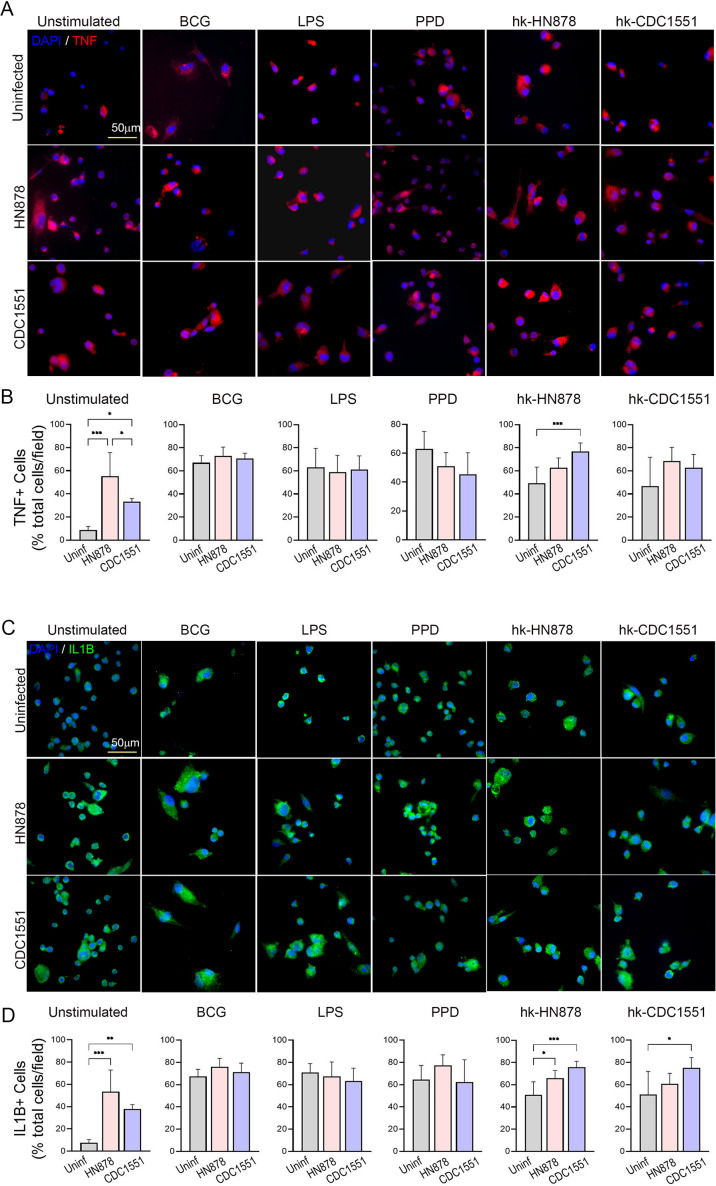
Spatial expression of proinflammatory markers in trained and restimulated macrophages with or without Mtb infection. Antibodies against TNF-α (**A and B**) and IL-1β (**C and D**) were used to determine the inflammatory response of trained and restimulated macrophages by immunocytochemistry analysis after 24 h of Mtb infection. Trained macrophages were restimulated with BCG, LPS, PPD, hk-HN878, or hk-CDC1551 or left without restimulation, and either infected with Mtb HN878 (HN878) or Mtb CDC1551 (CDC1551) or left uninfected (Uninfected) for 24 h. (**A**). Representative immunostaining image of macrophages showing expression of TNF-α (red spots). DAPI (blue) was used as a nuclear stain. The scale bar is 50 microns. (**B**). The percentage of TNF + cells among the total number of cells per field in trained macrophages with or without restimulation using BCG, LPS, PPD, hk-HN878, or hk-CDC1551, and infected with Mtb HN878 (HN878) or Mtb CDC1551 (CDC1551) or left without infection (Uninf). (**C**). Representative immunostaining image of trained and restimulated macrophages showing expression of IL-1β (green spots). DAPI (blue) was used as a nuclear stain. The scale bar is 50 microns. (**D**). The percentage of IL-1β + cells among the total number of cells per field in trained macrophages with or without restimulation using BCG, LPS, PPD, hk-HN878 or hk-CDC1551, and infected with Mtb HN878 (HN878) or Mtb CDC1551 (CDC1551) or left without infection (Uninf). For (**B and D**), random fields (*n* = 25 fields per sample; 2–3 samples per group) were microscopically analyzed at 63× magnification, and the total, as well as signal-positive cells, were manually counted. The data shown are the average ± standard deviation of 2–3 samples per group, repeated in duplicate. Statistical analyses were performed using one-way ANOVA with Tukey’s multiple-group comparison. *P < 0.05; **P < 0.01; ***P < 0.005; ****P < 0.001.

Next, we determined whether the expression of trained immunity markers was reflected at the individual cell level, we performed spatial imaging of trained and restimulated macrophages with or without subsequent Mtb infection, using immunocytochemistry analysis and enumerated the number of cells positive for TNF-α (TNF-α+) and IL-1β (IL-1β+) expression (Fig. 3). Compared to the controls (trained cells without restimulation), macrophages trained and restimulated with BCG, LPS, PPD, hk-HN, or hk-CDC showed an increased percentage of TNF-α+ and IL-1β + cells in the absence of Mtb infection (Fig. 3; left column). In the control macrophages, infection by both Mtb HN878 and CDC1551 upregulated the percentage of TNF-α + and IL-1β + cells (Fig. 3; middle and right columns). However, training/restimulation with BCG, LPS, or PPD did not show any significant difference in the percentage of TNF-α + and IL-1β + cells between Mtb-infected and uninfected macrophages. By contrast, hk-HN trained/restimulated macrophages showed a significantly higher percentage of TNF-α+ cells upon Mtb CDC1551 infection, and a significantly higher percentage of IL-1β + cells after infection with Mtb HN878 or CDC1551, compared to uninfected cells. Similarly, hk-CDC-trained/restimulated macrophages showed a significant increase in the percentage of IL-1β + cells after Mtb CDC1551 infection (Fig. 3). Thus, consistent with the gene expression analysis by qPCR (Fig. 1 and 2), the spatial expression analysis of trained/restimulated macrophages shows stimulant-dependent variations in the expression of key immune activation markers, which is further differentially regulated upon subsequent infection with divergent Mtb strains.

### Antimicrobial response of trained and restimulated macrophages

**Fig 4:**
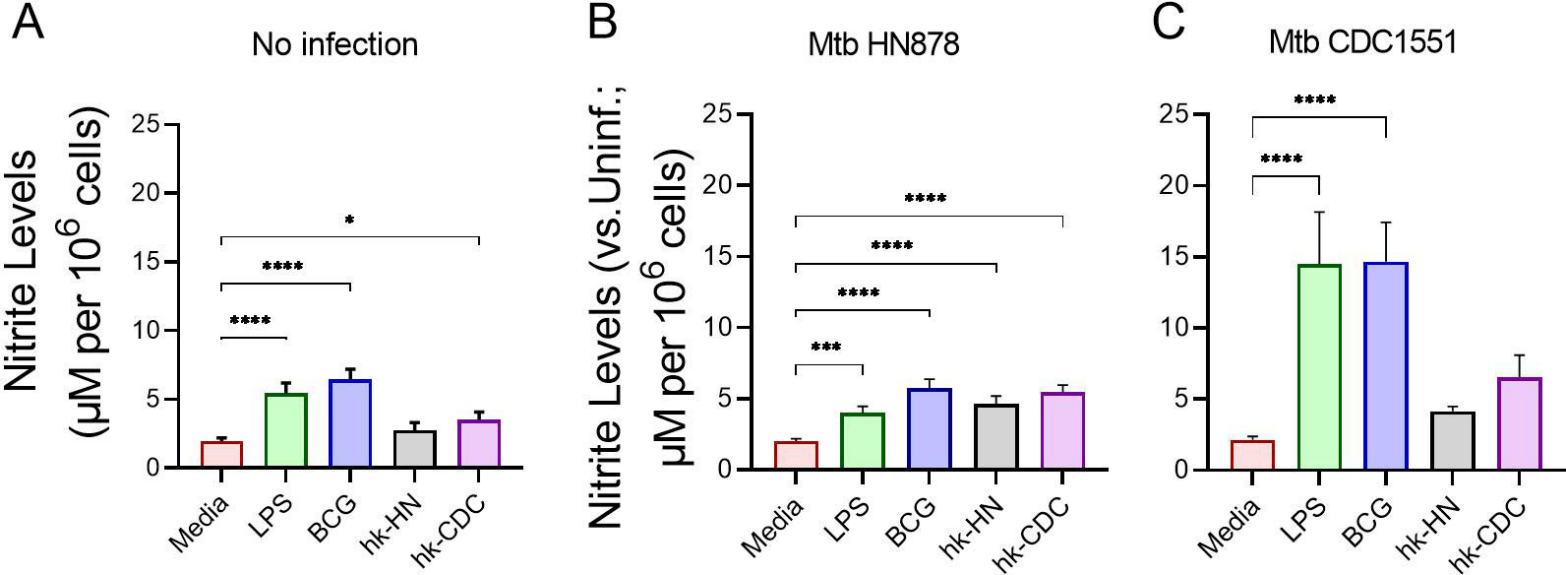
Antimycobacterial responses of trained and restimulated macrophages. Trained and restimulated macrophages were either infected with Mtb HN878 or CDC1551 or left untreated for 24 h. Cell-free supernatants were collected 24 h post-infection, and the nitrite levels were measured. (**A**) Trained and restimulated macrophages without infection. (**B**) Trained and restimulated macrophages infected with Mtb HN878. (**C**) Trained and restimulated macrophages infected with Mtb CDC1551. For (**A**), supernatants from only trained cells (without restimulation) were used as control (Media). For (**B and C**), the nitrite level in the uninfected macrophages (Uninf) was used to calibrate the corresponding levels in the Mtb-infected samples. The data shown are the average of three independent experiments performed in duplicates and the average of three experiments was used for plotting the graph. Statistical analyses were performed using one-way ANOVA with Tukey’s multiple-group comparison. *P < 0.05; **P < 0.01; ***P < 0.005; ****P < 0.001.

The antimicrobial response of macrophages upon activation is marked by elevated expression of iNOS (a.k.a. NOS2) with a corresponding increase in NO production (34, 75, 76). To determine the antimicrobial response of macrophages trained/restimulated with BCG, LPS, PPD, hk-HN, or hk-CDC, we measured the levels of NO production and *NOS2* expression (Fig. 4). Compared to the control (training only), training/restimulation with LPS, BCG, or hk-CDC significantly induced the NO production in uninfected macrophages (Fig. 4A). Infection of the trained and restimulated macrophages with Mtb HN878 also induced significantly more NO, compared to the uninfected controls, irrespective of the stimulant used for training/restimulation (Fig. 4B). However, Mtb CDC1551 infection significantly increased the NO production only in LPS or BCG-trained/restimulated macrophages (Fig. 4C). Thus, NO levels in trained and restimulated macrophages indicate variable activation of antimicrobial response upon infection with different types of Mtb strains.

### Immunometabolic shift in trained and restimulated macrophages

**Fig 5:**
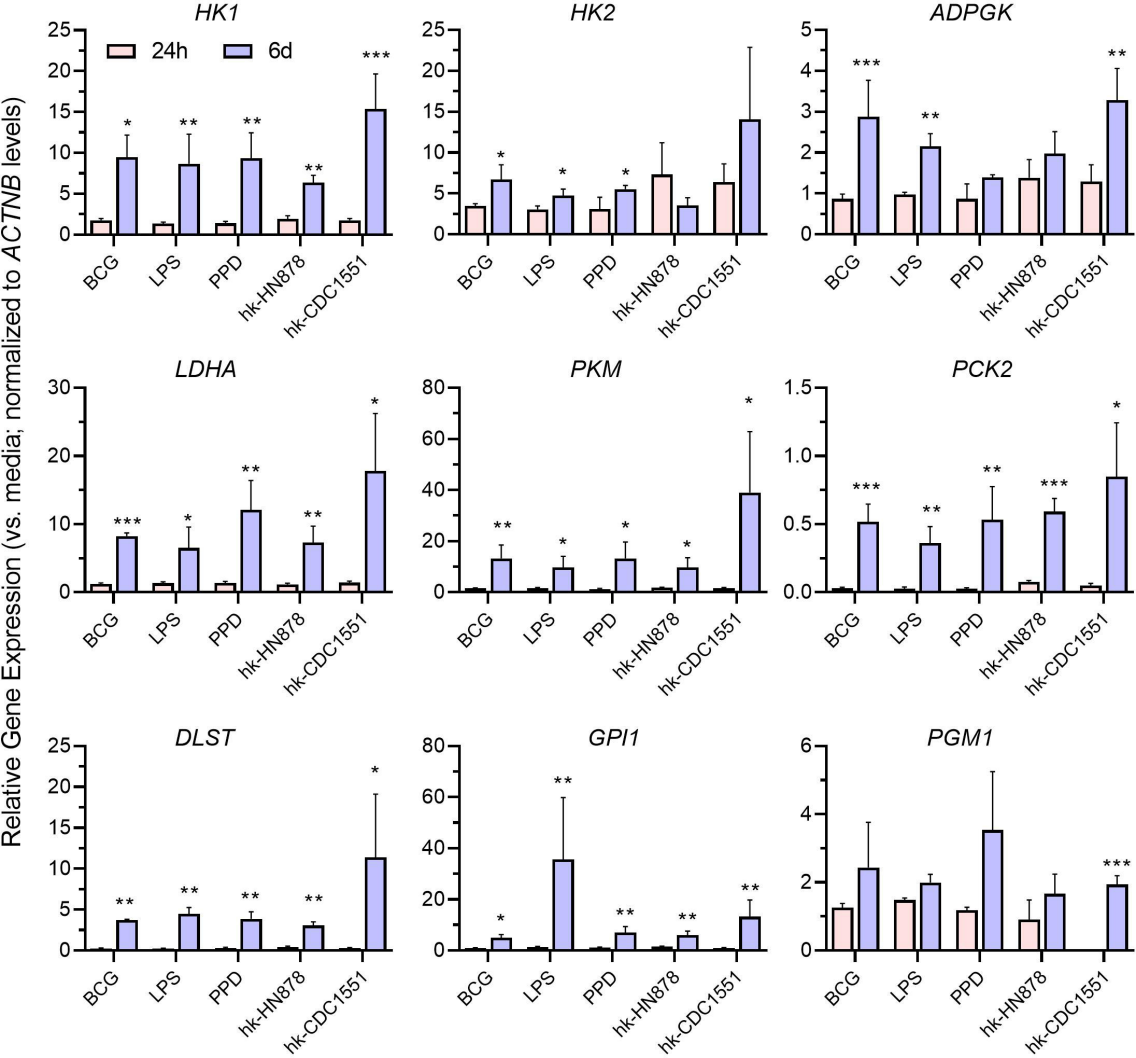
Expression profile of metabolic genes in trained and restimulated macrophages. Expression levels of host cell metabolic genes *HK1*, *HK2*, *ADPGK*, *LDHA, PKM*, *PCK2, GPI*, *PGM1,* and *DLST* were analyzed after training (24 h) or restimulation (6 d) of macrophages. Target gene expression was normalized to the *ACTNB* (beta-actin) expression levels in corresponding samples. The expression level of test genes in the unstimulated (media-control) samples was used to calibrate levels in trained samples with or without restimulation. The data shown are the average of three independent experiments performed in duplicates and the average of three experiments was used for plotting the graph. Statistical analyses were performed using one-way ANOVA with Tukey’s multiple-group comparison. *P < 0.05; **P < 0.01; ***P < 0.005.

Previous reports show that training of macrophages with BCG or LPS is associated with immunometabolic changes toward induction of glycolysis (77). However, the immunometabolic changes of trained macrophages following restimulation with other stimulants, such as PPD, hk-HN, or hk-CDC remain unknown. We tested the immunometabolic status of macrophages trained and restimulated with BCG, LPS, PPD, hk-HN, or hk-CDC by gene expression analysis and metabolite measurements (Fig. 5). The list of metabolic genes tested includes glycolysis (*HK1*, *HK2*, *ADPGK*, *LDHA, GPI*, *PGM1*, and *PKM*), tricarboxylic acid (TCA) cycle (*DLST*), and gluconeogenesis (*PCK2*). Expression of most of the tested glycolysis genes was significantly upregulated in trained macrophages (i.e., after 24 h of first stimulation), compared to the untrained cells with any of the stimulants (Fig. S5). However, expression of the TCA cycle gene was significantly downregulated upon primary stimulation (training), compared to unstimulated controls. Compared to the primary stimulated cells, the trained and restimulated macrophages consistently and significantly upregulated the expression of the tested TCA cycle gene, (Fig. 5). By contrast, the genes of glycolysis/gluconeogenesis showed a mixed response; while expression of GPI1 was significantly upregulated in macrophages trained/restimulated with all tested stimulants, PGM1 showed a significant upregulation only in macrophages trained/restimulated with hk-CDC (Fig. 5). These results suggest a metabolic activation toward glycolysis and TCA cycle in trained and restimulated macrophages, compared to training alone.

**Fig 6:**
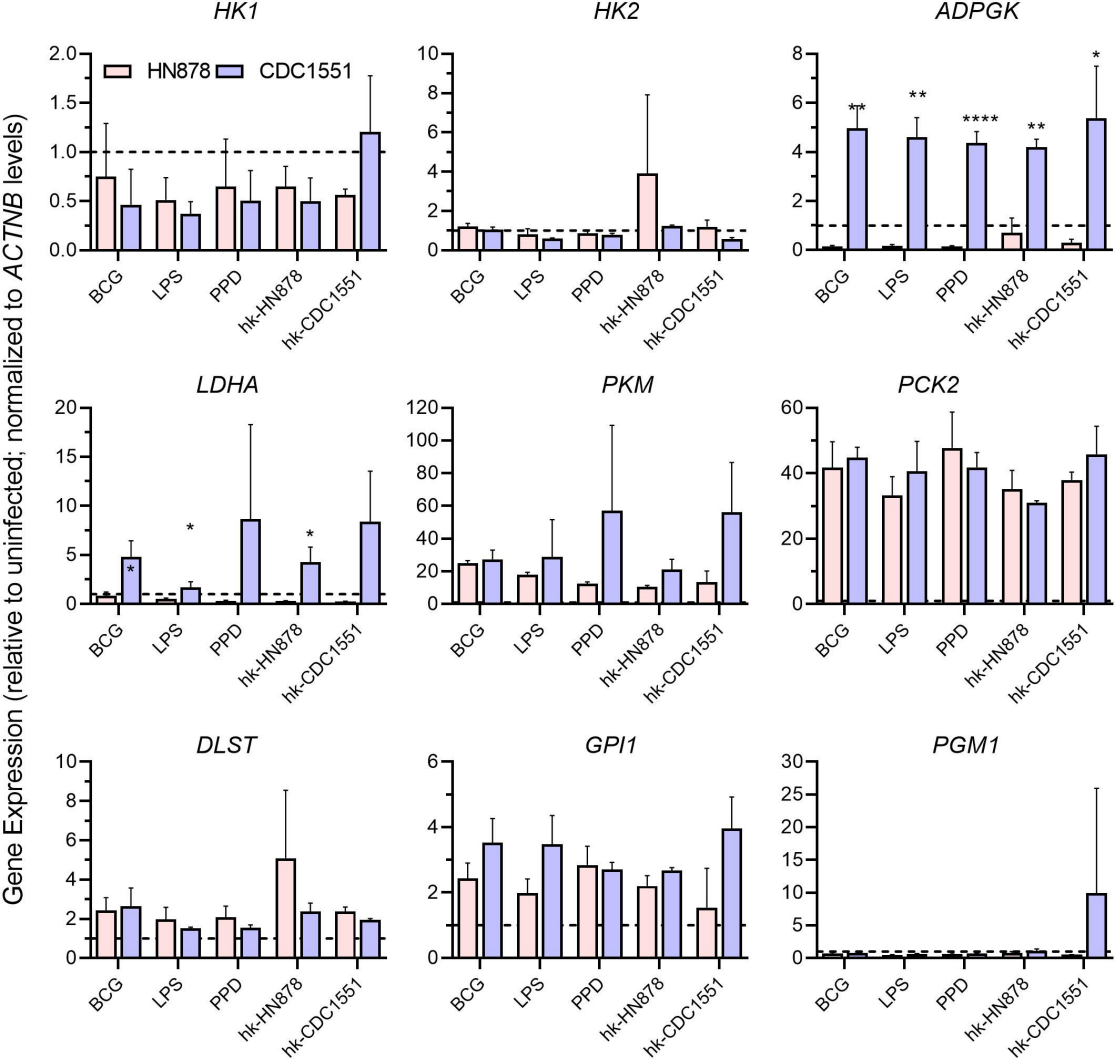
Expression profile of metabolic genes in trained and restimulated macrophages infected with Mtb. Expression levels of host cell metabolic genes *HK1*, *HK2*, *ADPGK*, *LDHA, PKM*, *PCK2, GPI*, *PGM1,* and *DLST* were analyzed in trained and restimulated macrophages after infection with clinical Mtb isolates HN878 or CDC1551. Target gene expression was normalized to the *ACTNB* (beta-actin) expression levels in corresponding samples. The expression level of test genes in the trained but not restimulated samples (dotted lines) was used to calibrate levels in the trained and stimulated samples. Gene expression values shown for Mtb-infected cells are relative to uninfected levels. The data are from an average of three independent experiments performed in duplicates. The data shown are the average of three independent experiments performed in duplicates and the average of three experiments was used for plotting the graph. Statistical analyses were performed using one-way ANOVA with Tukey’s multiple-group comparison. *P < 0.05; **P < 0.01; ****P < 0.001.

Next, we evaluated the changes in the expression of these metabolic markers in trained/restimulated macrophages after infection with Mtb strains, HN878 or CDC1551 (Fig. 6). Compared to the uninfected controls, the HN878-infected trained/restimulated macrophages did not show any significant changes in the expression of glycolysis genes except PKM and GPI1, although the TCA cycle and gluconeogenesis genes were significantly upregulated. By contrast, Mtb CDC1551 infection significantly upregulated the expression of selective markers of glycolysis (*ADPGK* and *LDHA*) and all tested TCA cycle and gluconeogenesis genes, compared to the uninfected controls (Fig. 6). These observations suggest Mtb-strain-specific metabolic alterations, particularly in the glycolysis and TCA cycle pathway, during infection of trained and restimulated macrophages.

**Fig 7:**
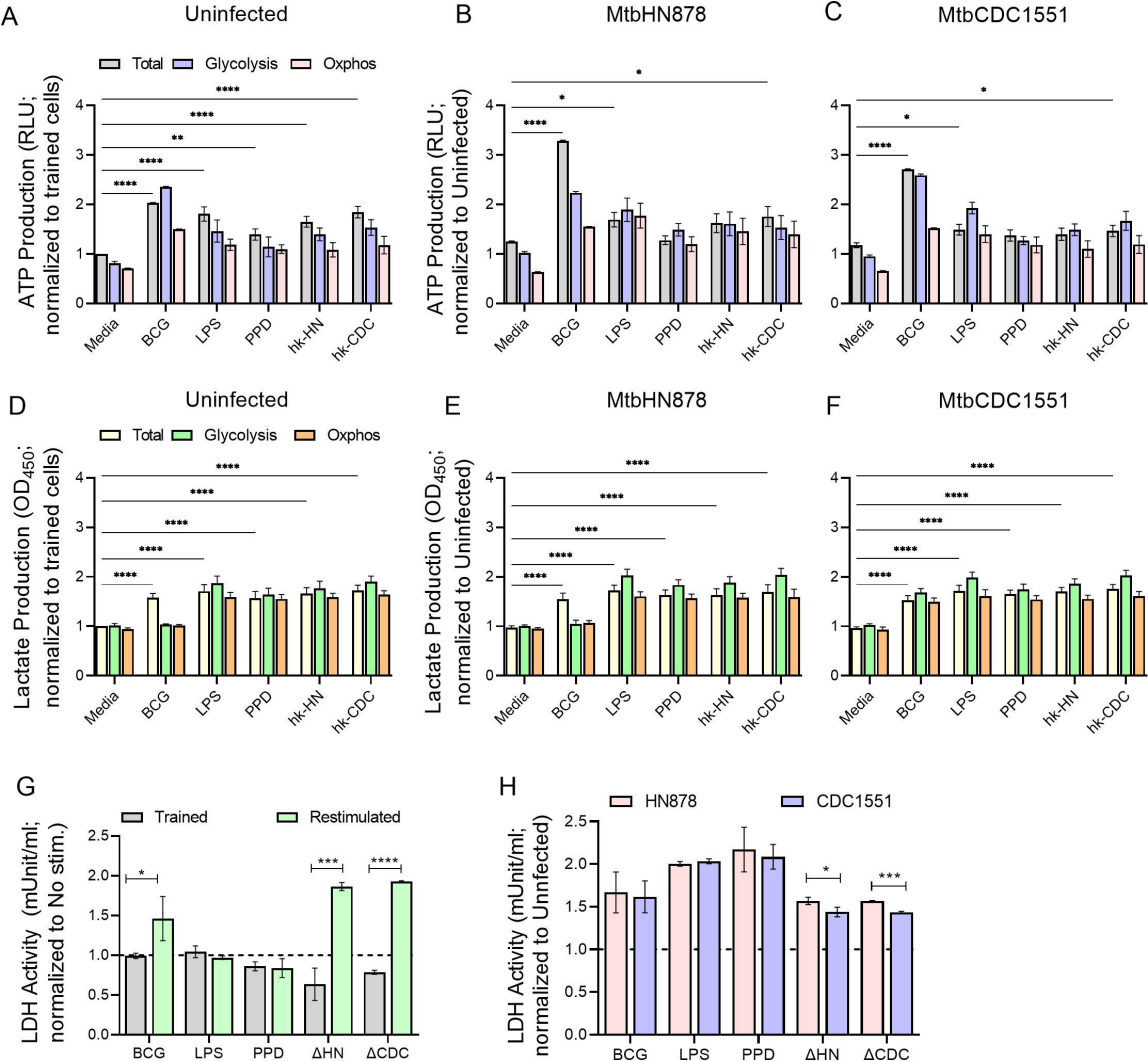
Production of ATP, lactate, and LDH activity in trained and restimulated macrophages with or without Mtb infection. Trained macrophages with or without restimulation were analyzed for total (Total), glycolytic (Glycolysis), and oxidative phosphorylation (Oxphos)-mediated ATP production in uninfected (**A**) or Mtb HN878-infected (**B**) or Mtb CDC1551-infected (**C**) conditions. In the same experimental condition, the total (Total), glycolytic (Glycolysis), and oxidative phosphorylation (Oxphos)-mediated lactate production were measured in uninfected (**D**), Mtb HN878-infected (**E**), or Mtb CDC1551-infected (**F**) macrophages. For (**A–F**), Oligomycin and 2-DG were used to inhibit ATP synthesis mediated through Oxphos and glycolysis, respectively. (**G**). LDH activity was measured in trained macrophages with or without restimulation with various stimulants, compared to no stimulation controls (dotted line). (**H**). LDH activity in MtbHN878 or MtbCDC1551-infected, trained, and restimulated macrophages, relative to uninfected cells (dotted line). The delta symbol (Δ) refers to heat-killed bacteria. The data shown are average ± standard deviations of three independent experiments performed in duplicates. Statistical analyses were performed using one-way ANOVA with Tukey’s multiple-group comparison. *P < 0.05; **P < 0.01; ***P < 0.005; ****P < 0.001.

Furthermore, we determined the contribution of metabolic pathways to the cellular ATP and lactate production in uninfected but trained and restimulated macrophages, compared to trained cells without restimulation (Fig. 7). The macrophages trained and restimulated with BCG, PPD, LPS, hk-HN, or hk-CDC showed significantly increased total ATP levels, compared to untrained (media) cells (Fig. 7A). Similarly, the glycolytic ATP and oxphos ATP levels were also higher in the trained/restimulated macrophages. However, there was no significant difference between glycolytic and oxphos ATP levels, suggesting that both pathways were contributing comparatively to the total ATP levels in the trained/restimulated macrophages (Fig. 7A).

Next, we tested the effect of Mtb infection on the metabolic marker expression in trained/restimulated macrophages, compared to uninfected cells. Although Mtb infection elevated the total, glycolytic and oxphos ATP production by the trained/restimulated macrophages, irrespective of the nature of the infecting strain, BCG-trained/restimulated macrophages had significantly more total ATP levels upon both Mtb HN878 and CDC1551-infection, compared to the other stimulant-treated trained macrophages (Fig. 7B and C). In contrast to the uninfected trained/restimulated macrophages, the Mtb-infected cells had higher activation of glycolysis and oxphos. However, no striking differences were noted in the total, glycolytic, and oxphos-mediated lactate production between the uninfected and Mtb-infected macrophages trained and restimulated with various stimulants (Fig. 7D through F).

Finally, the activity of the LDH enzyme was measured in the cell-free supernatants of untrained and trained and stimulated macrophages with or without Mtb infection (Fig. 7G and H). Compared to the trained cells, training and restimulation of macrophages with BCG, hk-HN, or hk-CDC significantly increased the LDH activity. In these trained/restimulated cells, Mtb infection further significantly upregulated the LDH activity, compared to the uninfected controls. However, Mtb HN878 infection showed a significantly higher LDH activity, compared to CDC1551 infection in hk-HN and hk-CDC trained and restimulated macrophages (Fig. 7G and H).

Together, the immunometabolic analyses indicate an overall elevation in the metabolic activation of trained and restimulated macrophages, compared to the trained cells without restimulation, although the extent of such activation and the distinct pathways impacted are driven by specific stimulants that train these cells. Furthermore, in these trained/restimulated macrophages with differential metabolic modulation, Mtb infection differentially perturbs the metabolic activation, in a strain-dependent manner.

### Regulation of autophagy in trained and restimulated macrophages with or without Mtb infection

**Fig 8:**
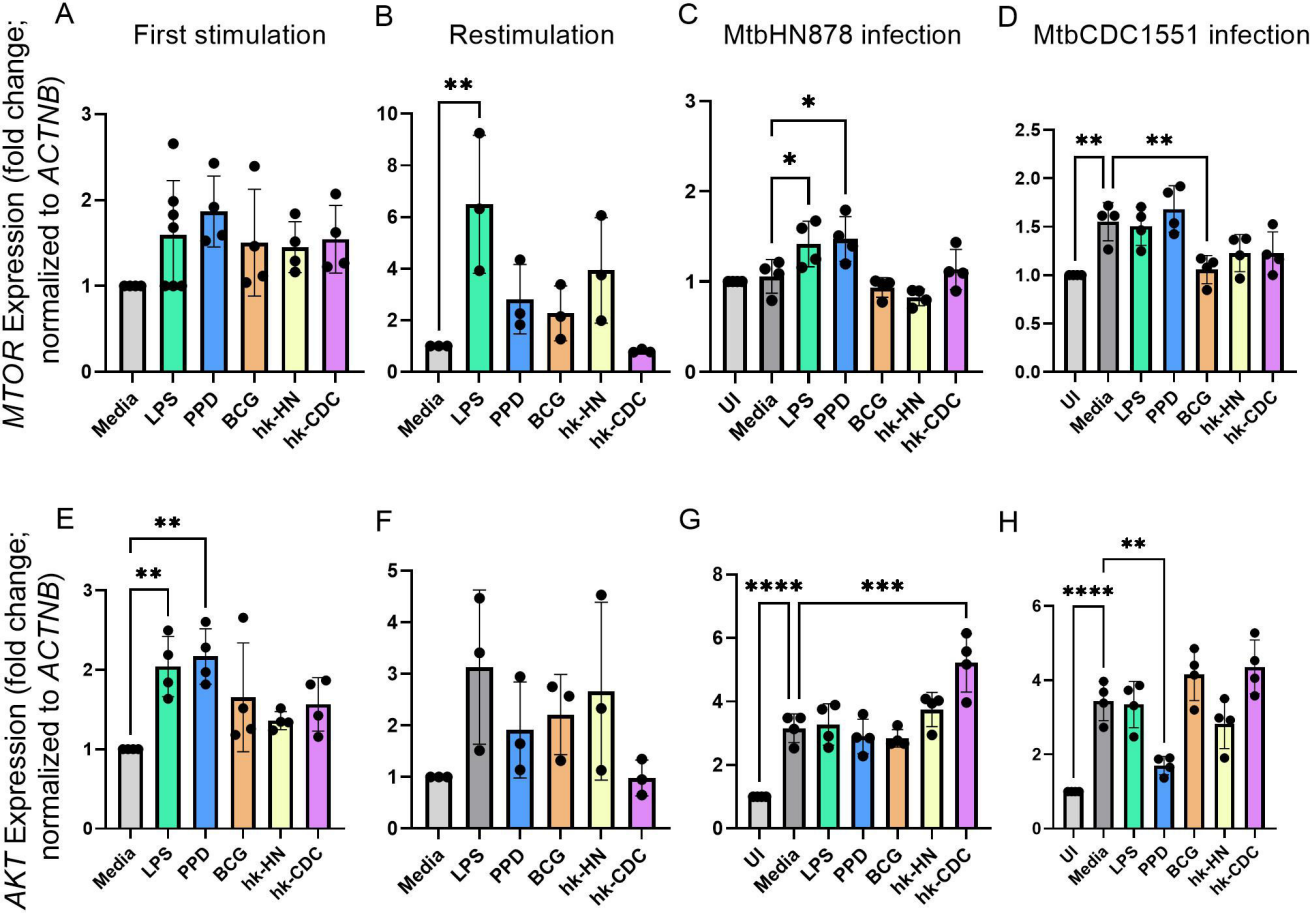
Differential expression of key autophagy regulator genes in trained and restimulated macrophages. Expression levels of autophagy regulator *mTOR* were measured by real-time PCR in trained (**A**) or trained and restimulated (**B**) macrophages without any infection (**A, B**) or after infection with Mtb HN878 (**C**) or Mtb CDC1551 (**D**). Similarly, the expression of another key autophagy regulator *AKT1* was analyzed in trained (E) or trained and restimulated (**F**) macrophages without any infection (**E, F**) or after infection with Mtb HN878 (**G**) or Mtb CDC1551 (**H**). The data shown are the average ± standard deviation of three independent experiments repeated twice. Statistical analyses were performed using one-way ANOVA with Tukey’s multiple-group comparison. *P < 0.05; **P < 0.01; ***P < 0.005; ****P < 0.001.

Autophagy has been reported as an immune activation mechanism of BCG-trained macrophages (78, 79). Here, we analyzed the expression levels of key autophagy regulatory genes *MTOR* and *AKT1*. Both these genes were upregulated in macrophages trained with various stimulants, compared to untrained cells (without stimulation) (Fig. 8A and E). Importantly, restimulation of these trained macrophages further elevated the expression of *MTOR* and *AKT1* compared to trained macrophages (media; without restimulation) (Fig. 8B and F). Furthermore, a significant upregulation of *MTOR* expression was noted upon Mtb HN878 and not CDC1551 infection of the macrophages trained and restimulated with LPS or PPD (Fig. 8C and D). Similarly, a significant upregulation in the expression of *AKT* was noted in Mtb HN878 and not CDC1551 infection of the macrophages trained and restimulated with hk-CDC (Fig. 8G and H). By contrast, expression of MTOR and AKT was significantly downregulated in Mtb CDC1551-infected macrophages trained and restimulated with BCG or PPD, respectively (Fig. 8D and H).

**Fig 9:**
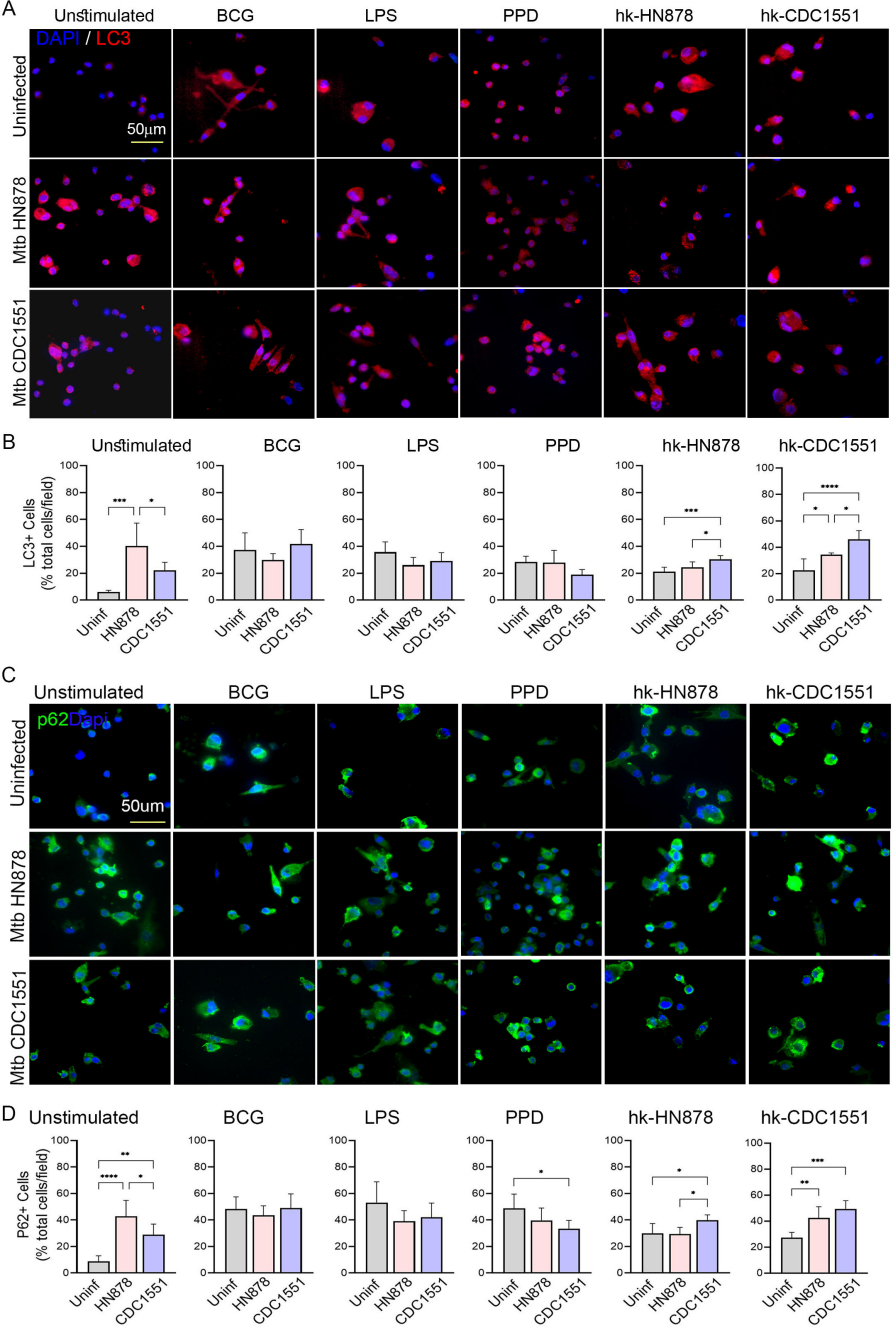
Spatial analysis of autophagy induction markers in trained and restimulated macrophages with or without Mtb infection: Antibodies against LC3 and P62 were used to determine the autophagy induction in macrophages by immunocytochemistry analysis. Trained and restimulated macrophages, as well as trained cells without restimulation (Unstimulated) were either infected with Mtb HN878 or Mtb CDC1551 or left uninfected (Uninfected) and images were taken 24 h post-infection. (**A**). Representative images of macrophages showing expression of autophagic marker LC3 (red spots). DAPI (blue) was used as a nuclear stain. The scale bar is 50 microns. (**B**). The percentage of LC3+ cells among the total number of cells per field in trained macrophages with or without restimulation. (**C**). Representative images of macrophages showing expression of autophagic marker P62 (green spots). DAPI (blue) was used as a nuclear stain. The scale bar is 50 microns. (**D**) The percentage of P62+ cells among the total number of cells per field in trained macrophage with or without restimulation. For (**B and D**), random fields (*n* = 25 fields per sample; 2–3 samples per group) were microscopically analyzed at 63× magnification, and the total, as well as signal-positive cells, were manually counted. The data shown are the average ± standard deviation of 2–3 samples per group repeated in duplicates. Statistical analyses were performed using one-way ANOVA with Tukey’s multiple-group comparison. *P < 0.05; **P < 0.01; ***P < 0.005; ****P < 0.001.

**Fig 10:**
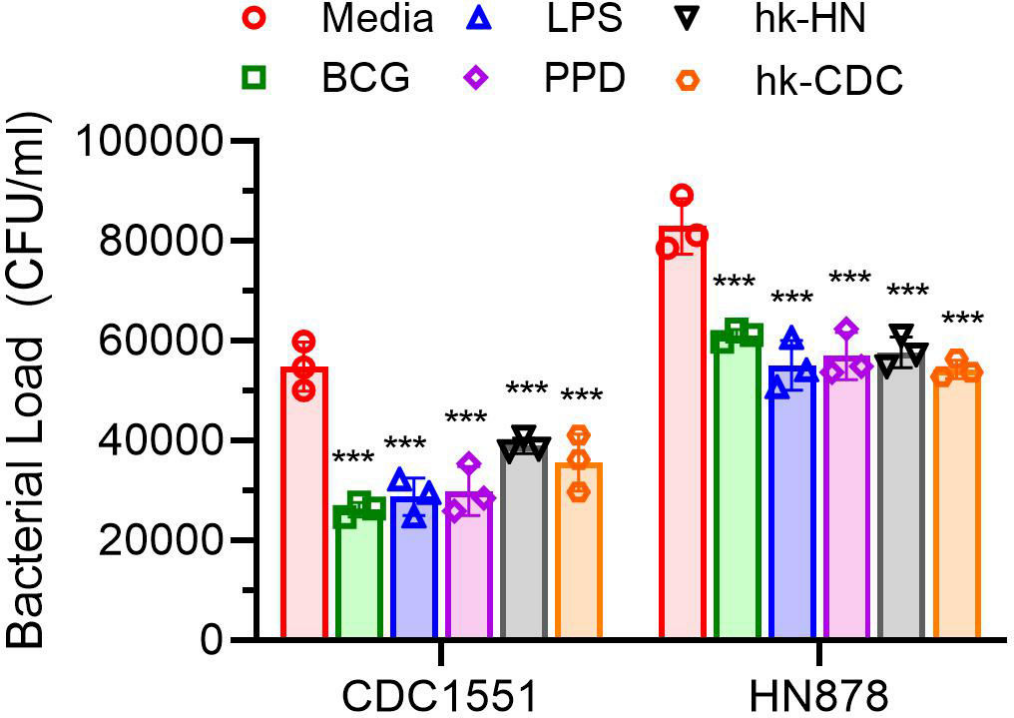
Intracellular survival of Mtb in trained and restimulated macrophages. Bacterial load was determined in trained and restimulated macrophages infected with Mtb HN878 or CDC1551 for 72 h. Cells were infected and lysed as described in the methods, spread on agar plates, and bacterial CFU was counted after 4–6 weeks of incubation of the plates. Lysates from trained cells (without restimulation) infected with Mtb were used as control (Media). The data shown are the average of three independent experiments performed in duplicates. The average of two technical replicates of each biological sample was used for plotting the graph. Statistical analyses were performed using one-way ANOVA with Tukey’s multiple-group comparison. *P < 0.05; **P < 0.01; ***P < 0.005; ****P < 0.001.

To further confirm the differential induction of autophagy in trained macrophages with or without subsequent Mtb infection, we performed a spatial immunocytochemistry analysis of macrophages using antibodies against P62/SQSTM1 and LC3, which are specific markers of autophagy (80, 81). As shown in Fig. 10, infection by both Mtb HN878 and CDC1551 significantly upregulated the percentage of P62+ and LC3+ macrophages in unstimulated conditions (Fig. 9A and B). The trained and restimulated macrophages showed a higher percentage of P62 and LC3 positivity, compared to the unstimulated cells (Fig. 9A; left column). However, Mtb HN878 and CDC1551 infection differentially impacted these autophagic marker expressions in the trained and restimulated macrophages. The PPD or LPS-trained and restimulated macrophages had fewer P62+ and LC3+ cells upon infection by Mtb HN878 or CDC1551, although the reduction of P62+ cells was significant only for CDC1551 infection in PPD-trained macrophages. By contrast, hk-HN-trained macrophages had a significantly higher percentage of P62+ and LC3+ cells after infection by both Mtb strains, compared to the uninfected cells. A similar response was noted for Mtb-infected macrophages trained with hk-CDC. This suggests that autophagy induction could involve other mechanisms that do not directly impact the PI3K/AKT/mTORC1 pathway. Further study is required to decipher the signaling pathway leading to the induction of autophagy in trained and restimulated macrophages.

### Restimulation augments the antimicrobial response of trained macrophages

Since restimulation of trained macrophages showed more robust immune activation, metabolic and autophagic responses, we tested the antimicrobial response of these macrophages, compared to trained macrophages without restimulation. Trained macrophages with or without restimulation were infected with Mtb HN878 or CDC1551, and the intracellular bacterial load was measured at 72 h post-infection by enumerating the number of bacterial CFU (Fig. 10). Compared to the trained cells, the LPS, BCG, PPD, hk-HN, or hk-CDC-trained and restimulated macrophages significantly reduced the intracellular bacterial load of both HN878 and CDC1551. The reduction in bacterial CFUs in these infected cells was comparable between various stimulants tested. Although the overall intracellular bacterial burden was higher in Mtb HN878-infected macrophages, the difference was not significantly different compared to the Mtb CDC1551-infected cells (Fig. 10). These observations suggest that elevated production of immune activation markers, including cytokines and NO production by macrophages trained and restimulated with various stimulants, endows these cells to control intracellular bacterial burden significantly, compared to trained macrophages without restimulation.

To delineate the impact of restimulation on trained macrophages in the context of Mtb infection, we evaluated the intracellular mycobacterial load in trained (but not restimulated) macrophages. We observed a significantly lower bacterial load in the trained macrophages, compared to the untrained cells (Fig. S6). However, the extent of bacterial load reduction was higher in the trained and restimulated macrophages, compared to the trained cells (compare Fig. 10; Fig. S6). These findings suggest that restimulation further elevates the antimicrobial responses of trained macrophages.

Finally, we compared the effectiveness of heterologous versus homologous stimulants in inducing autophagy and affecting the antimicrobial response of trained and restimulated macrophages (Fig. S7). These macrophages were trained and restimulated with different combinations of antigens and infected with pathogenic Mtb H37Rv, a standard laboratory strain. Everolimus (EVR) was used as an autophagy inducer. The number of intracellular bacteria in the trained and restimulated macrophages was enumerated after 72 h of infection in the presence or absence of EVR. As shown in Fig. S7, the intracellular bacillary load was significantly reduced in BCG and/or LPS or hk-Mtb-trained/restimulated macrophages in the presence of EVR, compared to no EVR-treatment group, suggesting a synergistic effect of autophagy induction in trained and restimulated macrophages against Mtb survival, although it was significantly affected only in a selected combination of heterologous antigens (Fig. S7).

## DISCUSSION

Trained immunity or innate immune memory refers to the ability of innate immune cells, such as monocytes and macrophages, to establish long-term immunity upon exposure to antigens that confer response to subsequent infections or stimuli (82). The primary study that laid the foundation of the trained immunity concept was carried out in mice. It was found that a single dose of *Candida albicans* provided a protective immune response against unrelated reinfection with another pathogen *Staphylococcus aureus* (83). Detailed *in vitro* analyses of the human monocyte infection model showed that β-glucan, the cell wall component of *C. albicans*, induced epigenetic remodeling and functional reprogramming of the host cells through a dectin-1/Raf1-dependent pathway (25). Importantly, β-glucan administration conferred protection against sepsis development during experimental *S. aureus* infection in a murine model (84). These trained innate immune cells showed increased production of hallmark pro-inflammatory cytokines, including TNF-α, IL-6, and IL-1β, as well as antimicrobial molecules such as reactive oxygen species (25, 84, 85). Thus, several studies have documented the response of macrophages after exposure to one stimulus or signal, which augments robust activation to a second signal, such as a bacterial infection (82–86). However, there is limited information available now regarding the immune response of macrophages repeatedly stimulated with the same stimulus, in the context of enhancing the capacity of these cells to counter subsequent infection. In this study, we used a homogeneous stimulation model, wherein the macrophages were trained, and then restimulated with the same/homogeneous stimulant. We found that the nature of antigen shapes the immunometabolic and cellular responses of stimulated macrophages, which differentially impacts the host response to subsequent Mtb infection. To assess the effectiveness of the enhanced immune response, we trained and restimulated the macrophages with the same stimulant so that the immune response would be maximized and render cells more capable of eliminating the invading pathogen.

Training of human monocytes with mycobacterial cell wall components, β-glucan, or muramyl dipeptide, as stimulants led to an enhanced cytokine and chemokine response upon subsequent Mtb infection (15, 87–89). However, exposure to other stimuli, such as LPS or peptidoglycan, did not have the same effect, suggesting that the specific nature of the antigen can influence the trained immunity response (90). Overall, these studies suggest that both types of stimulants, related and unrelated to a pathogen, can establish trained immunity in human monocytes/macrophages and that the magnitude and specificity of the response may vary depending on the nature of the antigen. Furthermore, the antigenicity of the stimuli is crucial for training the macrophages to gain an innate memory phenotype. Consistent with these findings, we noticed that homologous training and restimulation with BCG and hk-Mtb can also trigger a robust proinflammatory response in macrophages, compared to training and restimulation with LPS or PPD. However, the specific mechanisms underlying the differential response elicited by various stimulants, including the role of epigenetic modifications and other factors, remain to be determined.

Trained immunity in human macrophages is characterized by enhanced cytokine and chemokine responses as well as metabolic changes upon subsequent immune challenges (57). The specific cytokines and chemokines produced by these trained macrophages in response to different stimuli can vary depending on the nature and timing of the immune challenge (83, 91–93). For example, exposure of THP-1 macrophages to microbial cell walls components, such as β-glucan or muramyl dipeptide (MDP), led to increased production of a broad range of pro-inflammatory cytokines and chemokines including IL-1β, IL-6, TNF-α, IFN-γ, IL-8, and MCP-1 upon subsequent stimulation with various microbial stimuli, including LPS, a component of gram-negative bacterial cell walls (89). These cytokines play important roles in the innate immune response by inducing inflammation and recruiting other immune cells to the site of infection. We observed elevated production of IL-1β, IL-8, and TNF-α, particularly in macrophages trained and restimulated with BCG and hk-Mtb.

Macrophage activation and associated metabolic changes are important steps in determining the outcomes and the fate of pathogenic invasion of these cells (94, 95). It has been reported that BCG stimulation of macrophages leads to a shift of cellular metabolism from oxidative phosphorylation to glycolysis with increased expression of mTOR/Akt/HIF-1A pathway and elevated NO production (15, 77, 91, 93, 96–98). Similar to these reports, we found elevated immune activation marker expression, with concomitant induction of glycolysis and TCA cycle gene expression, increased ATP and lactate production as well as NO production in macrophages that were repeatedly stimulated with different stimuli. Although the LDH levels we observed in this study are contributed by Mtb-infected cells with or without apoptosis and/or necrosis, it is also possible that the stimulants we have used to train the macrophages might have contributed to LDH levels due to cell death. However, we did not measure the cell death due to various stimulants in this study.

Autophagy is a cellular process that plays a critical role in the maintenance of cellular homeostasis and the elimination of intracellular pathogens. Training of THP-1 macrophages with β-glucan or DMP and LPS led to increased expression of autophagy-related genes and autophagy flux as well as increased formation of autophagosomes and autolysosomes (15, 91, 96–98). In this study, we found autophagy induction, through elevated levels of autophagosome proteins (P62, LC3), to various extents in trained and restimulated macrophages, dependent upon the nature of the antigen used for stimulation. However, the question of whether autophagy induction in our studies by repeated stimulation with different stimuli (i.e., BCG, LPS, PPD, hk-HN, and hk-CDC) involves the same pathway as seen in classically trained macrophages needs additional mechanistic studies.

Previous studies have linked autophagy, antimicrobial response, immune activation, and killing of Mtb in THP-1 macrophages trained with various stimuli (65, 79). The ability of THP-1 cells trained with β-glucan, MDP, or BCG to effectively control intracellular Mtb growth was associated with increased autophagy in these cells, marked by elevated expression of several autophagy-related genes such as ATG5, LC3B, and p62 (65, 79). In addition, pharmacological inhibition of autophagy partially reversed the enhanced killing of Mtb by trained THP-1 cells, suggesting that autophagy plays a critical role in the trained immunity response (65, 78). The p62/SQSTM1 protein plays a regulatory role in the ubiquitin-proteasome system, cellular metabolism, signaling, and apoptosis, in addition to the classical autophagy activation pathway (99). Indeed, upregulation of p62 has been previously reported to promote autophagy, and depletion of p62 reduced autophagy by downregulation of ATG10 in cells exposed to oxidative stress (100). Similarly, although increased p62 levels under cell-damaging conditions have been reported to denote autophagy deficiency, the accumulation of p62 has been considered to have a cytoprotective response, particularly in atherosclerosis (101). Furthermore, the p62 expression level does not always inversely correlate with the level of autophagy; in muscle atrophy induced by cancer, autophagic stimuli induce the expression of p62 at gene and protein levels (102, 103). Thus, the role of p62 in autophagy is regulated by several context-dependent factors. In this study, we observed autophagy induction and p62 upregulation in homogeneously trained and restimulated macrophages. However, understanding the causal link between p62 upregulation and autophagy induction in the context of repeated stimulation in macrophages requires further mechanistic studies. Nonetheless, our findings suggest that trained THP-1 macrophages can display enhanced autophagy response to subsequent immune stimulation, which may play an important role in the elimination of intracellular pathogens and the maintenance of cellular homeostasis. However, the specific mechanisms underlying this effect upon repeated stimulation, including the role of autophagy-related genes, on the macrophage response to homologous training by various stimuli remain to be determined.

Trained immunity has also been shown to enhance the ability of THP-1 cells to kill clinical isolates of Mtb. A study by Kleinnijenhuis et al. showed that THP-1 cells trained with β-glucan or BCG led to an enhanced ability to control Mtb growth through increased production of TNF-α and IL-1β, as well as increased phagocytic activity (63, 104, 105). Similarly, we found a significant reduction in the growth of pathogenic Mtb clinical isolates HN878 and CDC1551, within trained and restimulated macrophages. Consistent with this observation, we found differential induction of NO, an anti-mycobacterial molecule, in trained and restimulated macrophages upon infection with these Mtb strains. However, there was no direct proportionality between the levels of cytokines and/or NO production by trained cells versus the number of intracellular CFUs in macrophages trained with various stimulants. This could be explained as either (i) there is a threshold level for NO-mediated Mtb killing or (ii) Mtb resists NO-mediated killing after a certain degree of death as a population in the macrophages. However, these assumptions need to be tested and validated by additional experimental studies. Furthermore, we showed previously that induction of autophagy is associated with elevated Mtb killing by macrophages (106). To determine whether induction of autophagy in repeatedly stimulated macrophages would augment Mtb killing, we induced autophagy in repeatedly trained macrophages by Everolimus treatment and analyzed the effect on intracellular Mtb survival. We found a deleterious effect on Mtb survival in repeatedly stimulated macrophages treated with Everolimus, indicating that evoking autophagy in this setting would benefit the host by effectively clearing the pathogen. Together, induction of NO production and autophagy are potential mechanisms for the antimicrobial response of macrophages that were trained and restimulated with different stimuli, although the extent of induction of these antimicrobial responses and bacterial killing are shaped by the nature of the antigen and the infecting Mtb strain.

In previous studies using *in vitro* (mouse and human primary cells and cell lines) and *in vivo* (mouse and rabbit) models of infection, we have demonstrated divergent, Mtb-strain-specific host immune responses to HN878 (a hypervirulent strain) and CDC1551 (a hyper immunogenic strain) (34, 73). In the present study, we show that macrophages trained and restimulated with various stimuli can enhance Mtb killing through several mechanisms, including autophagy and NO production as well as through the activation of cytokine, chemokine, and metabolic signaling pathways. Although these responses were reported in “classically trained” macrophages (without restimulation), our study provides additional information that restimulation of trained macrophages with different stimuli can mount a stronger immune response. These findings are significant since it signifies the use of different stimuli or a different combination of stimuli to boost immunity in macrophages, and possibly in other innate immune cells, to protect the host from various microbial infections. Since repeated stimulation of innate cells with multiple antigens substantially boosts host immunity, this strategy should be considered for developing better and improved vaccines to combat infectious diseases. Our studies have some limitations, such as the under-exploration of specific signaling mechanisms associated with the immune response in repeatedly stimulated macrophages, and the usage of a macrophage cell line rather than primary cells or *in vivo* models. Also, we did not titrate various doses of primary and secondary stimulants on their ability to mount trained immunity. Apart from these, we have not investigated the details of the role of specific cell death pathways, such as apoptosis, in trained cells in countering mycobacterial infection. Our study also lacks molecular mechanisms, like plasma membrane orchestration involved in the entering of Mtb inside macrophages and the fusion of Mtb-containing phagosomes with lysosomes in infected cells.

In summary, our study highlights the intricate host cell adaptations during innate memory responses, including immunometabolic shift elicited by various stimulants, and how these responses impact subsequent antimicrobial effects of macrophages to infection by pathogenic Mtb strains. The findings of this study can pave the way to develop better vaccines against TB, by endowing the innate immune cells with improved antimicrobial and protective immune responses through training. Similarly, some of the innate memory response pathways can be harnessed as targets to develop host-directed therapy (HDT) to enhance bacterial clearance from infected organs and/or to alleviate host damage caused by Mtb infection.

## Data Availability

All the experimental data are presented as figures and tables in this manuscript. Raw data of the figures and tables can be obtained from the lead contact upon formal request. This article does not report any original code.

## SUPPLEMENTAL MATERIAL

Figure S1 spectrum.00473-24-s0001.docx Experimental setup for macrophage training and restimulation and Mtb infection.

Figure S2 spectrum.00473-24-s0002.docx Cytokine profiling of trained macrophages.

Figure S3 spectrum.00473-24-s0003.docx Visualization of Mtb in infected trained and restimulated macrophages.

Figure S4 spectrum.00473-24-s0004.docx Expression profile of trained human macrophages.

Figure S5 spectrum.00473-24-s0005.docx Metabolic profile of trained THP-1 macrophages.

Figure S6 spectrum.00473-24-s0006.docx Differential responses of trained macrophages to Mtb infection.

Figure S7 spectrum.00473-24-s0007.docx Autophagy induction augments Mtb killing in trained and restimulated macrophages.

Table S1 spectrum.00473-24-s0008.docx List of target macrophage genes and the DNA sequence.

ASM does not own the copyrights to Supplemental Material that may be linked to, or accessed through, an article. The authors have granted ASM a non-exclusive, world-wide license to publish the Supplemental Material files. Please contact the corresponding author directly for reuse.
