# Supplementary material for: The innate memory response of macrophages to Mycobacterium tuberculosis is shaped by the nature of the antigenic stimuli
Source: Microbiol Spectr. 2024 Jul 9;12(8):e00473-24. doi: 10.1128/spectrum.00473-24 (PMC11302266; doi:10.1128/spectrum.00473-24)
Supplement: Figure S1 — Experimental setup for macrophage training and restimulation and Mtb infection. [file spectrum.00473-24-s0001.docx]

**Supplementary Figure 1**


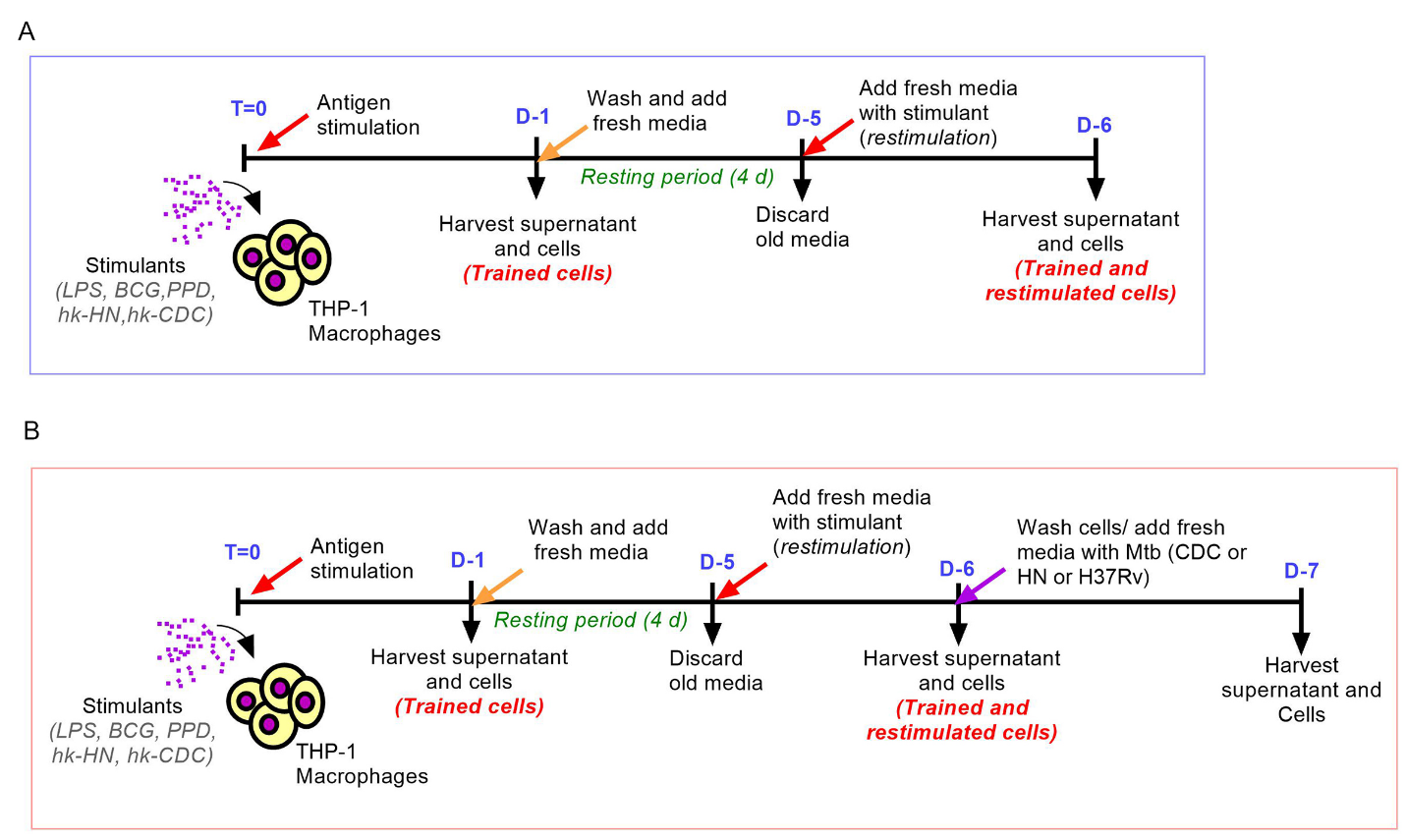


**Supplementary Figure 1. Experimental setup for macrophage training and restimulation and Mtb infection. A**.THP-1 derived macrophages were first stimulated with BCG, LPS, PPD, hk-HN878 or hk-CDC1551 (training). After 24h of stimulation (Day 1) the cells were washed and fresh media without any stimulants was added. The cells were left for 4 days (resting period). Media was removed on day 5 and fresh media containing the same antigens as the primary stimulation was added to the cells (restimulation). The cells were incubated for another 24 h (Day 6). After training (Day 1) and restimulation (Day 6), culture supernatants were collected, and cells were harvested for downstream analysis. Macrophages with only training (without restimulation) were used as controls. **B.** In a similar setting as in A, at the end of 2nd stimulation (Day 6), the macrophages were washed and incubated with fresh media containing Mtb HN878 or CDC1551 or H37Rv at MOI of 1 and incubated for 24h (Day 7). At the end of incubation, cell-free culture supernatants and cells were collected for downstream assays. Uninfected cells were used as a negative control.
