# Supplementary material for: The innate memory response of macrophages to Mycobacterium tuberculosis is shaped by the nature of the antigenic stimuli
Source: Microbiol Spectr. 2024 Jul 9;12(8):e00473-24. doi: 10.1128/spectrum.00473-24 (PMC11302266; doi:10.1128/spectrum.00473-24)
Supplement: Figure S2 — Cytokine profiling of trained macrophages. [file spectrum.00473-24-s0002.docx]

**Supplementary Figure 2**


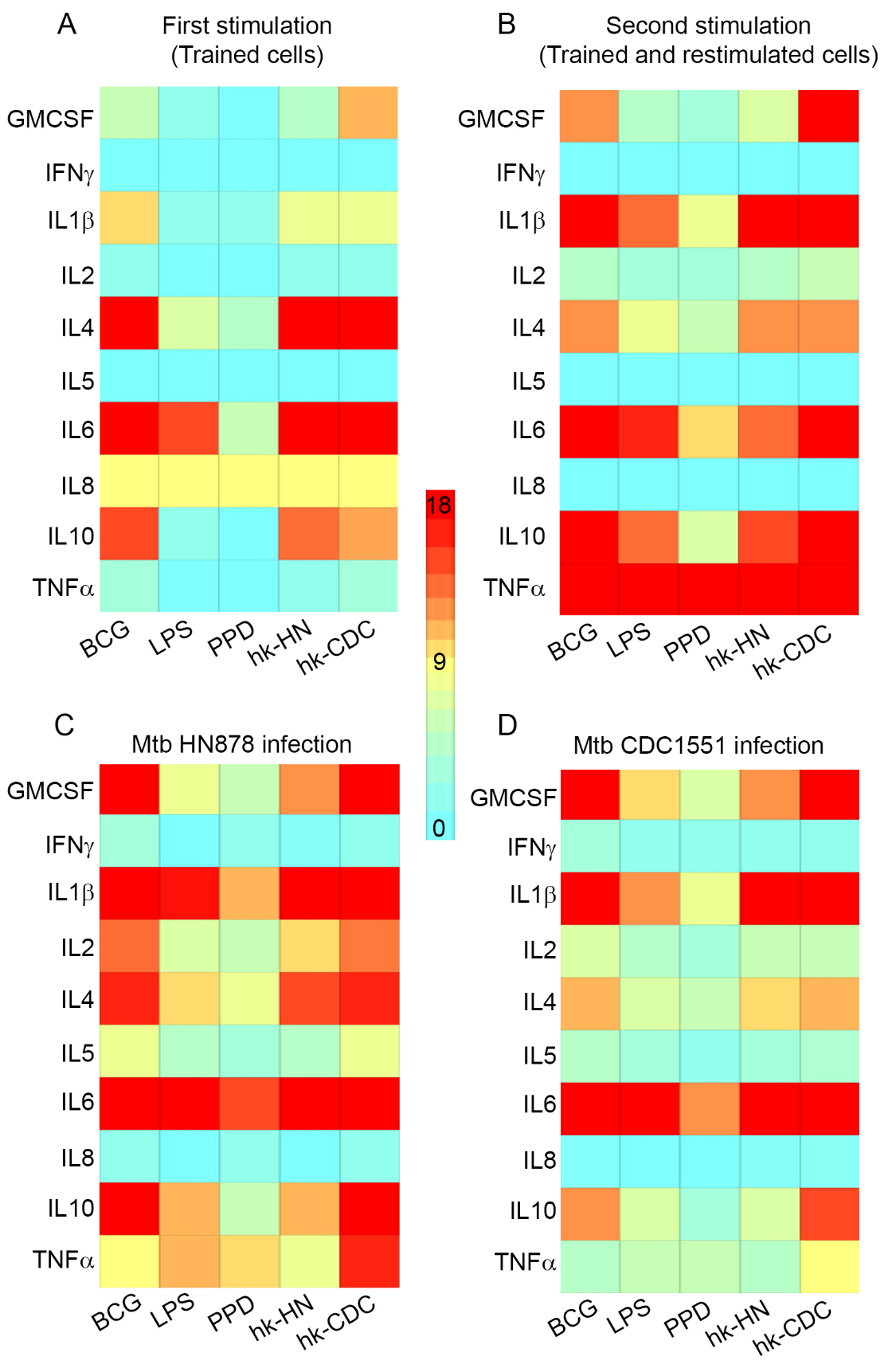


**Supplementary Figure 2.** **Cytokine profiling of trained macrophages with or without restimulation and with or without Mtb infection.** Cell-free culture supernatants were collected from trained (first stimulation) or trained and restimulated (second stimulation) macrophages without or with Mtb HN878 or Mtb CDC1551 infection. The protein levels of selected cytokines were determined by Luminex Multiplex Assay as described in the methods section. The amount of cytokines released is represented in the form of a heat map with a color coding bar indicating the level of expression (blue-low; red-high).
