# Supplementary material for: The innate memory response of macrophages to Mycobacterium tuberculosis is shaped by the nature of the antigenic stimuli
Source: Microbiol Spectr. 2024 Jul 9;12(8):e00473-24. doi: 10.1128/spectrum.00473-24 (PMC11302266; doi:10.1128/spectrum.00473-24)
Supplement: Figure S3 — Visualization of Mtb in infected trained and restimulated macrophages. [file spectrum.00473-24-s0003.docx]

**Supplementary Figure 3**


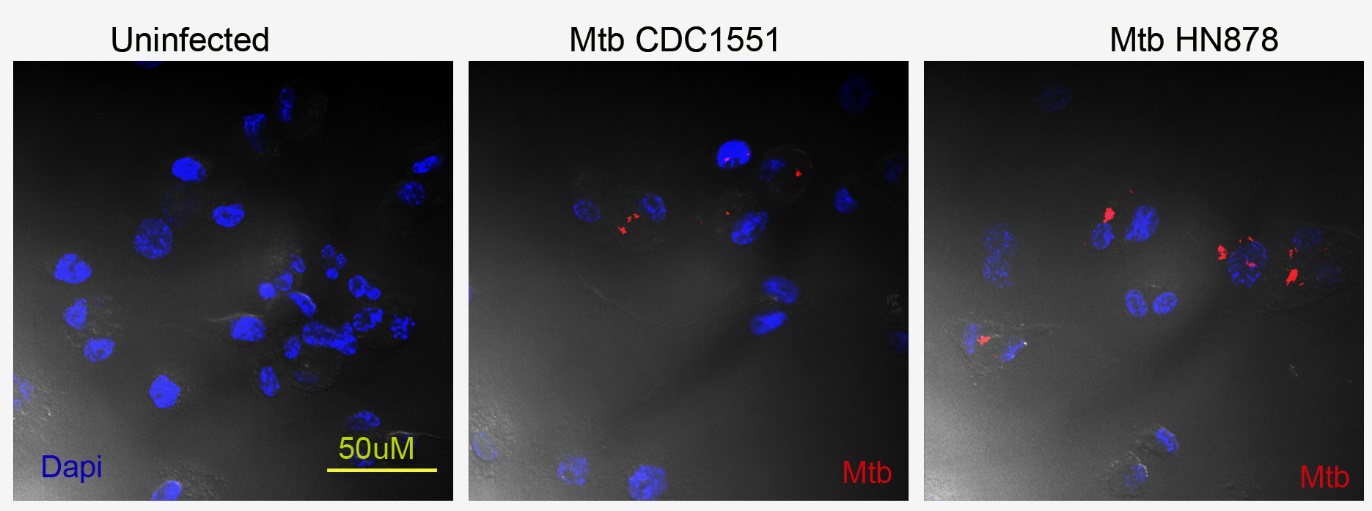


**Supplementary Figure 3. Visualization of Mtb in infected trained and restimulated macrophages.** The auramine-Rhodamine staining method was used to detect Mtb in infected macrophages (red color rods), as described in the methods. DAPI was used as a nuclear stain for the host cells (blue color). Stained cells were examined under a fluorescence microscope using a 63X objective and photographed. The scale bar is 50 µm.
