# Supplementary material for: The innate memory response of macrophages to Mycobacterium tuberculosis is shaped by the nature of the antigenic stimuli
Source: Microbiol Spectr. 2024 Jul 9;12(8):e00473-24. doi: 10.1128/spectrum.00473-24 (PMC11302266; doi:10.1128/spectrum.00473-24)
Supplement: Figure S4 — Expression profile of trained human macrophages. [file spectrum.00473-24-s0004.docx]

**Supplementary Figure 4**


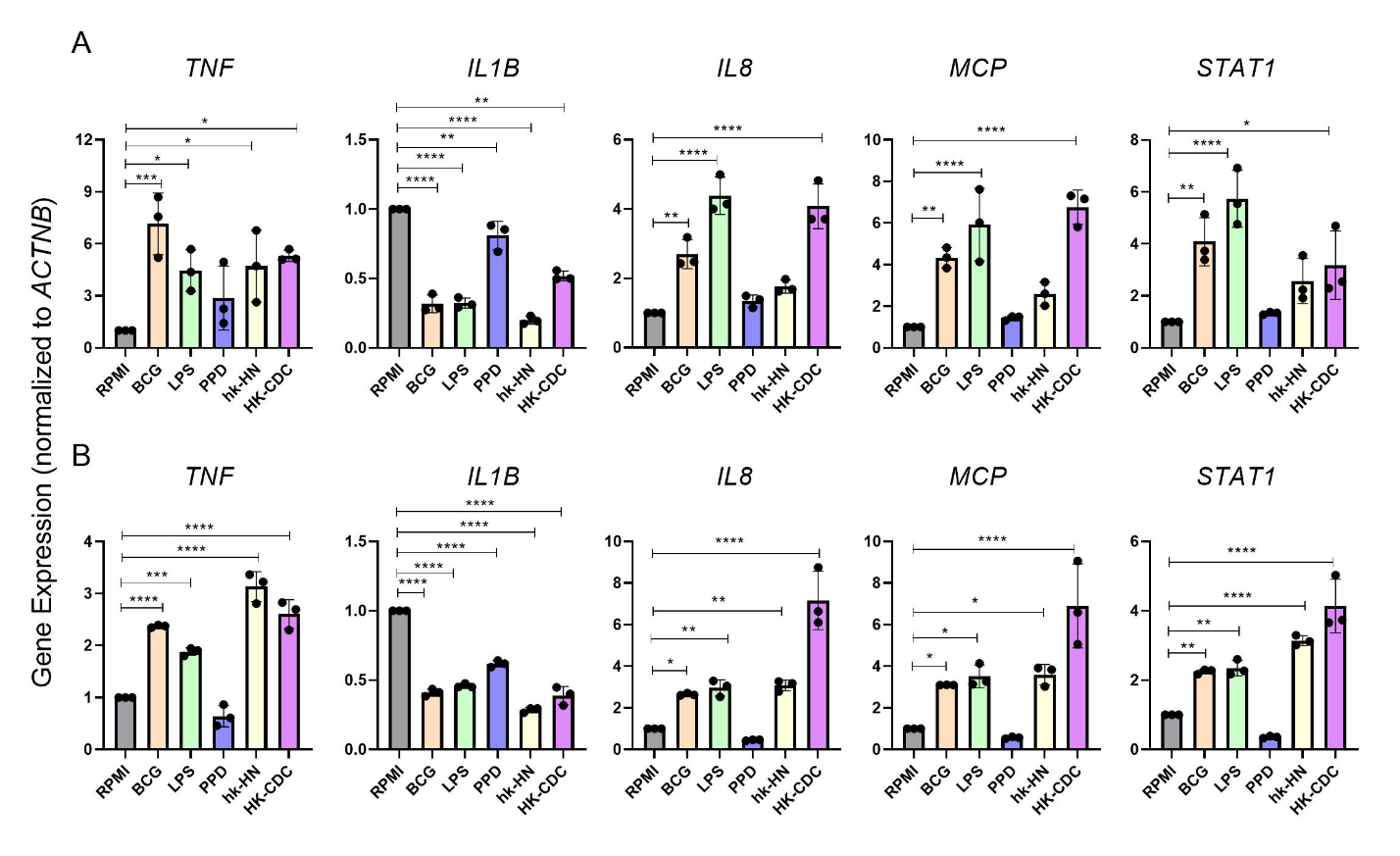


**Supplementary Figure 4. Expression profile of trained human macrophages after infection with clinical Mtb isolates.** THP-1 derived macrophages were stimulated for 24 h followed by resting for 4 days (classical training protocol) with BCG, LPS, PPD, hk-HN878 or hk-CDC1551, and infected with clinical Mtb isolates, HN878 (**A**) or CDC1551 (**B**) for 24 h. Total RNA was isolated and used for qPCR analysis of genes encoding proinflammatory cytokines/chemokine/transcription factor (TNF-α, IL-1β, IL-8, MCP-1and STAT-1). RPMI refers to unstimulated (control) macrophages. Target gene expression was normalized to the *ACTB* expression levels in corresponding samples. The data shown are the average of three independent experiments performed in duplicates. The average of two technical replicates of each biological sample was used for plotting the graph. Statistical analyses were performed using one-way ANOVA with Tukey’s multiple-group comparison. *p < 0.05; ** p < 0.01; ***p < 0.005; **** p < 0.001.
