# Supplementary material for: The innate memory response of macrophages to Mycobacterium tuberculosis is shaped by the nature of the antigenic stimuli
Source: Microbiol Spectr. 2024 Jul 9;12(8):e00473-24. doi: 10.1128/spectrum.00473-24 (PMC11302266; doi:10.1128/spectrum.00473-24)
Supplement: Figure S5 — Metabolic profile of trained THP-1 macrophages. [file spectrum.00473-24-s0005.docx]

**Supplementary Figure 5**


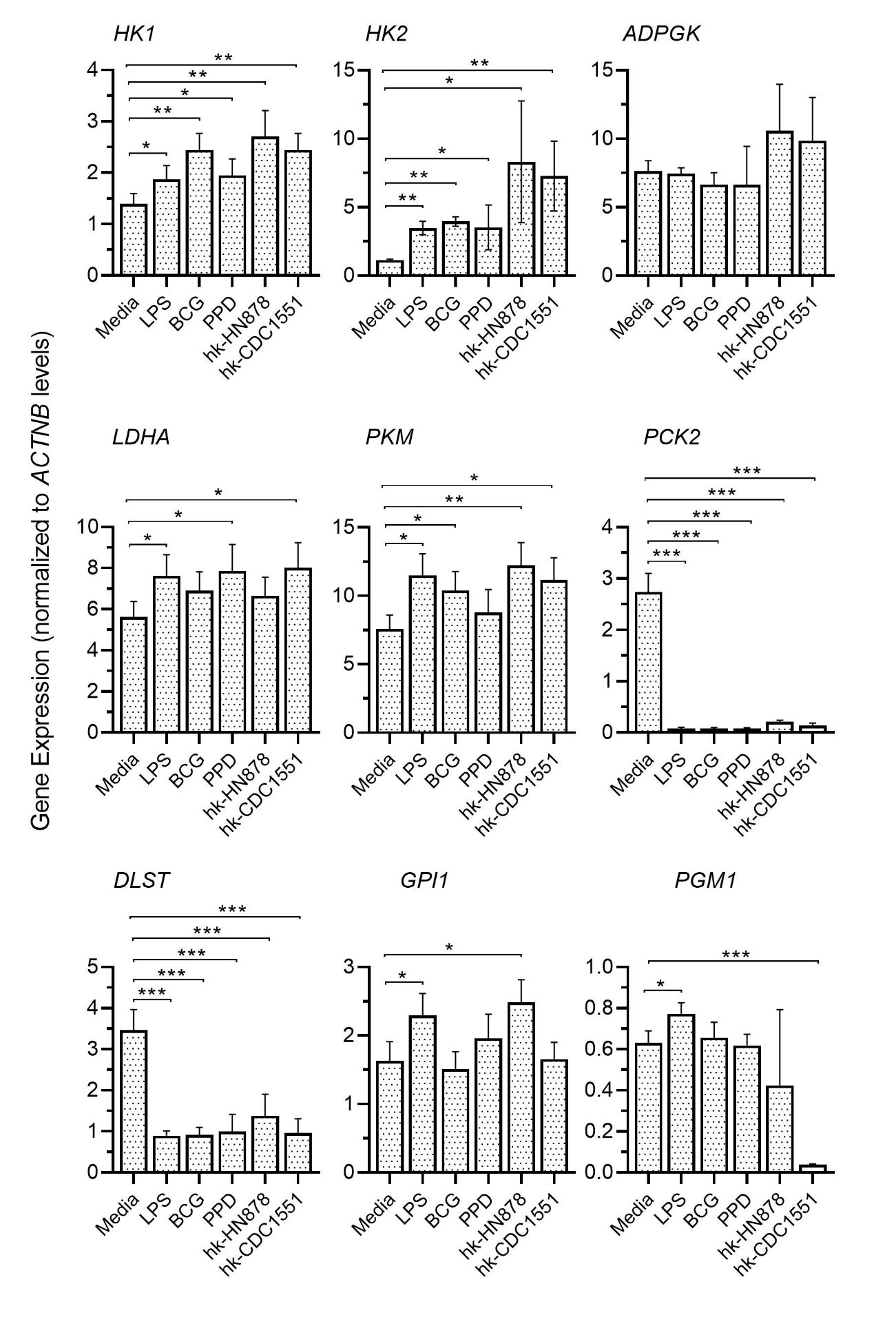


**Supplementary Figure 5.** **Metabolic profile of trained THP-1 macrophages.** Total RNA was isolated from macrophages trained with various antigens (BCG, LPS, PPD, hk-HN878 or hk-CDC1551) and used for qPCR analysis to assess the expression of selected metabolic genes, including *HK1*, *HK2*, *ADPGK*, *LDHA*, *PKM*, *PCK2*, *DLST*, *GPI* and *PGM1*. Media refers to unstimulated (control) macrophages. Target gene expression was normalized to the *ACTB* expression levels in corresponding samples. The data shown are the average of three independent experiments performed in duplicates. Statistical analyses were performed using unpaired Student’s t-test between untrained (media) and antigen-trained samples. *p < 0.05; ** p < 0.01; ***p < 0.005; **** p < 0.001.
