# Supplementary material for: The innate memory response of macrophages to Mycobacterium tuberculosis is shaped by the nature of the antigenic stimuli
Source: Microbiol Spectr. 2024 Jul 9;12(8):e00473-24. doi: 10.1128/spectrum.00473-24 (PMC11302266; doi:10.1128/spectrum.00473-24)
Supplement: Figure S6 — Differential responses of trained macrophages to Mtb infection. [file spectrum.00473-24-s0006.docx]

**Supplementary Figure 6**


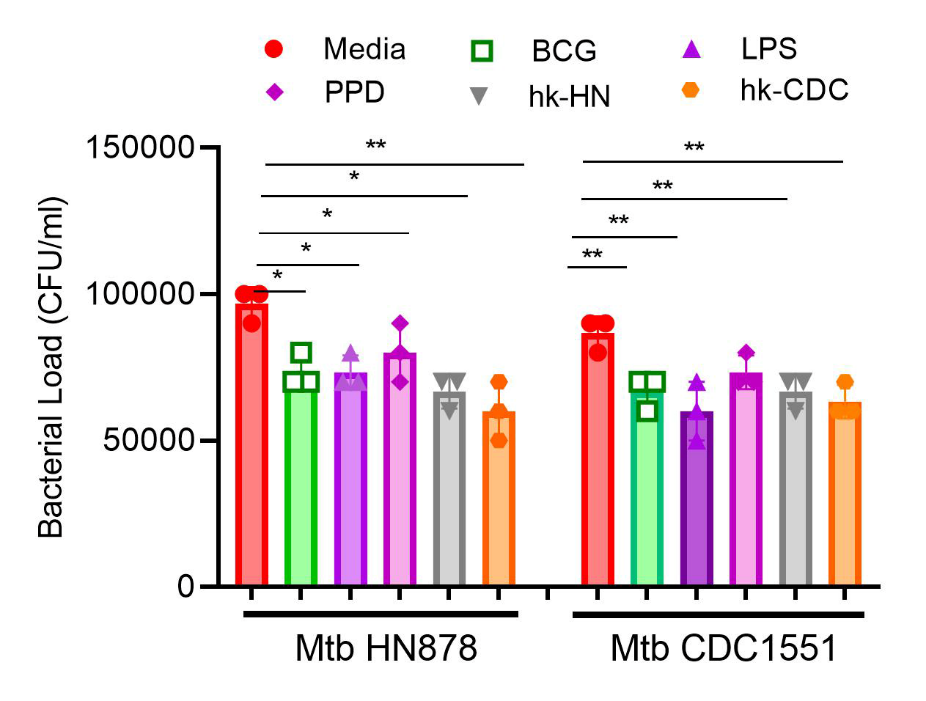


**Supplementary Figure 6. Differential responses of trained macrophages to Mtb infection.** THP-1-derived macrophages were trained (stimulation for 24h, followed by resting for 4 days) with various stimulants and either infected with Mtb HN878 or CDC1551 at MOI of 1 or left untreated for 24 hours. Cells were lysed as described in the methods section, spread on agar plates, and bacterial CFU was counted after 2 weeks of incubation of the plates. The data shown are the average of three independent experiments performed in duplicates. The average of two technical replicates of each biological sample was used for plotting the graph. Statistical analyses were performed using an unpaired Student’s t-test between media and antigen-stimulated samples. *p < 0.05; ** p < 0.01.
