# Supplementary material for: The innate memory response of macrophages to Mycobacterium tuberculosis is shaped by the nature of the antigenic stimuli
Source: Microbiol Spectr. 2024 Jul 9;12(8):e00473-24. doi: 10.1128/spectrum.00473-24 (PMC11302266; doi:10.1128/spectrum.00473-24)
Supplement: Figure S7 — Autophagy induction augments Mtb killing in trained and restimulated macrophages. [file spectrum.00473-24-s0007.docx]

**Supplementary Figure 7**


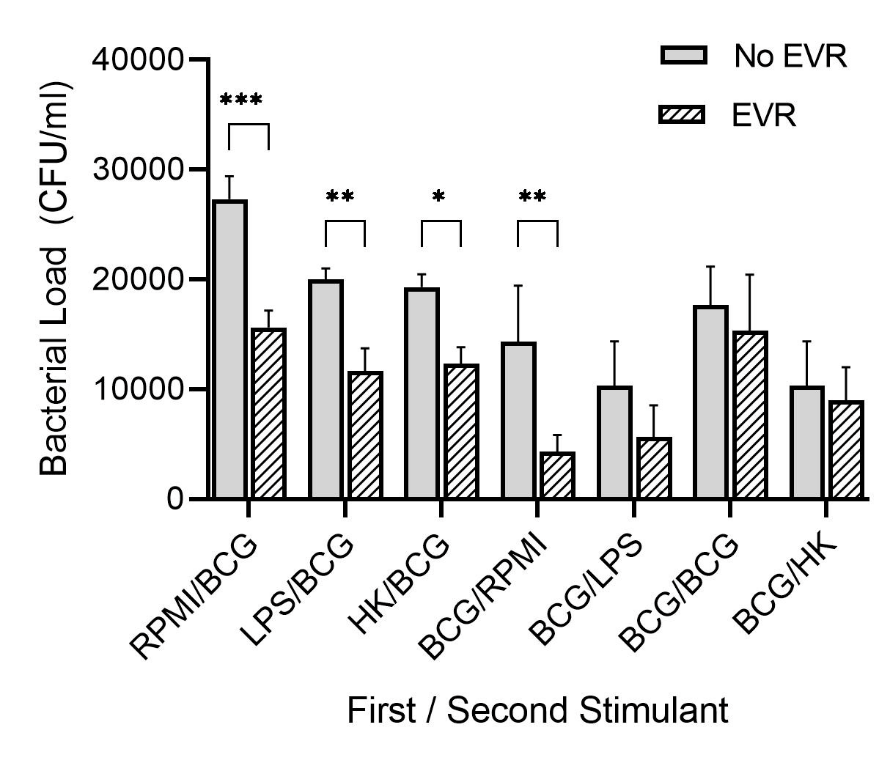


**Supplementary Figure 7: Autophagy induction augments Mtb killing in trained and restimulated macrophages.** THP-1-derived macrophages were trained and stimulated with various antigens as indicated, followed by infection with pathogenic Mtb H37Rv strain in the presence or absence of an autophagy inducer everolimus (EVR) as described in methods. Infected macrophages with or without EVR treatment were lysed and Mtb survival was assessed by CFU assay. The data shown are the average +/- standard deviation of three independent experiments performed in duplicates. Statistical analyses were performed using an unpaired T-test (EVR versus no EVR) with Welsh correction. *p < 0.05; ** p < 0.01; ***p < 0.005.
