# Supplementary material for: The innate memory response of macrophages to Mycobacterium tuberculosis is shaped by the nature of the antigenic stimuli
Source: Microbiol Spectr. 2024 Jul 9;12(8):e00473-24. doi: 10.1128/spectrum.00473-24 (PMC11302266; doi:10.1128/spectrum.00473-24)
Supplement: Table S1 — List of target macrophage genes and the DNA sequence. [file spectrum.00473-24-s0008.docx]

**Supplementary Table 1.** List of target genes and the DNA sequence of forward and reverse primers used in qPCR experiments.

| **Sr. no.** | **Target Gene** | **Primer Name** | **Sequence (5' -> 3')** |
| --- | --- | --- | --- |
| 1 | *TNFA* | Hu_TNF_F1 | CCTCTCTCTAATCAGCCCTCTG |
|  |  | Hu_TNF_R1 | GAGGACCTGGGAGTAGATGAG |
| 2 | *IL1B* | Hu_IL1B_F1 | GTCATTCGCTCCCACATTCT |
|  |  | Hu_IL1B_R1 | CTACTTCTTGCCCCCTTTGA |
| 3 | *IL8* | Hu_IL8_F1 | ACTGAGAGTGATTGAGAGTGGAC |
|  |  | Hu_IL8_R1 | AACCCTCTGCACCCAGTTTTC |
| 4 | *IL12A* | Hu_IL12A_F1 | CCTTGCACTTCTGAAGAGATTGA |
|  |  | Hu_IL12A_R1 | ACAGGGCCATCATAAAAGAGGT |
| 5 | *MCP1* | Hu_MCP1_F1 | CCAGTCACCTGCTGTTATAAC |
|  |  | Hu_MCP1_R1 | TGGAATCCTGAACCCACTTCT |
| 6 | *TLR2* | Hu_TLR2_F1 | TTATCCAGCACACGAATACACAG |
|  |  | Hu_TLR2_R1 | AGGCATCTGGTAGAGTCATCAA |
| 7 | *TLR4* | Hu_TLR4_F1 | AGACCTGTCCCTGAACCCTAT |
|  |  | Hu_TLR4_R1 | CGATGGACTTCTAAACCAGCCA |
| 8 | *STAT1* | Hu_STAT1_F1 | ATCAGGCTCAGTCGGGGAATA |
|  |  | Hu_STAT1_R1 | TGGTCTCGTGTTCTCTGTTCT |
| 9 | *IRF1* | Hu_IRF1_F1 | ATGCCCATCACTCGGATGC |
|  |  | Hu_IRF1_R1 | CCCTGCTTTGTATCGGCCTG |
| 10 | *HK1* | Hu_HK1_F1 | TCCTGATGGCTCTGAAAAGG |
|  |  | Hu_HK1_R1 | ACGATGTTCTCTGGGGTGTC |
| 11 | *HK2* | Hu_HK2_F1 | CAAAGTGACAGTGGGTGTGG |
|  |  | Hu_HK2_R1 | CTTCCGATTTCAGGGGTTCT |
| 12 | *ADPGK* | Hu_ADPGK_F1 | TATCAAGCAGGGGAGGAGTG |
|  |  | Hu_ADPGK_R1 | AATCCAGAGAGGACCACCAG |
| 13 | *LDHA* | Hu_LDHA_F1 | AGCCCGATTCCGTTACCT |
|  |  | Hu_LDHA_R1 | CAGAGAGACACCAGCAACATTC |
| 14 | *PKM* | Hu_PKM_F1 | TCCAGGTGAAGCAGAAAGGT |
|  |  | Hu_PKM_R1 | ACAGCAGGCAAGTCCACAG |
| 15 | *PCK2* | Hu_PCK2_F1 | GGCTGGAAAGTGGAGTGTGT |
|  |  | Hu_PCK2_R1 | GATTGGTGGTGGCAGAGGT |
| 16 | *DLST* | Hu_DLST_F1 | TCTGTGCAGGTTCCATCACC |
|  |  | Hu_DLST_R1 | AGTGTGAAAAGTGGAGTGCCT |
| 17 | *GPI1* | Hu_GPI1_F1 | AGAGAGGGGACAAGCAGGAC |
|  |  | Hu_GPI1_R1 | CCAAGCCCAAGGTGTGATAG |
| 18 | *PGM1* | Hu_PGM1_F1 | GGAATTTGATGGACGCGAGC |
|  |  | Hu_PGM1_R1 | TGGTGAAGAAATTCCGGCCA |
| 19 | *MTOR* | Hu_mTOR_F1 | TCCGAGAGATGAGTCAAGAGG |
|  |  | Hu_mTOR_R1 | CACCTTCCACTCCTATGAGGC |
| 20 | *AKT1* | Hu_AKT1_F1 | GCGGGGTAGGGAAGAAAAC |
|  |  | Hu_AKT1_R1 | TGACAGAGTGAGGGGACACA |
| 21 | *GAPDH* | Hu_GAPDH_F1 | CTGGGCTACACTGAGCACC |
|  |  | Hu_GAPDH_R1 | AAGTGGTCGTTGAGGGCAATG |
| 22 | *ACTNB* | Hu_ACTB_F1 | CATGTACGTTGCTATCCAGGC |
|  |  | Hu_ACTB_R1 | CTCCTTAATGTCACGCACGAT |
